# Tuning the properties of tris(hydroxypyridinone) ligands: efficient ^68^Ga chelators for PET imaging†
†Electronic supplementary information (ESI) available. See DOI: 10.1039/c8dt04454f


**DOI:** 10.1039/c8dt04454f

**Published:** 2019-03-12

**Authors:** Cinzia Imberti, Yu-Lin Chen, Calum A. Foley, Michelle T. Ma, Brett M. Paterson, Yifu Wang, Jennifer D. Young, Robert C. Hider, Philip J. Blower

**Affiliations:** a King's College London , School of Biomedical Engineering and Imaging Sciences , St Thomas’ Hospital , London SE1 7EH , UK . Email: Philip.Blower@kcl.ac.uk ; Email: Cinzia.Imberti@kcl.ac.uk; b King's College London , School of Biomedical Sciences , Institute of Pharmaceutical Science , London , SE1 9NH UK; c University of Melbourne , School of Chemistry , Melbourne , VIC 3010 , Australia

## Abstract

The prototype tris(1,6-dimethyl-3-hydroxypyridin-4-one) chelator for gallium-68, THP^Me^, has shown great promise for rapid and efficient kit-based ^68^Ga labelling of PET radiopharmaceuticals. Peptide derivatives of THP^Me^ have been used to image expression of their target receptors *in vivo* in preclinical and clinical studies. Herein we describe new synthetic routes to the THP platform including replacing the 1,6-dimethyl-3-hydroxypyridin-4-one N^1^–CH_3_ group of THP^Me^ with O (tris(6-methyl-3-hydroxypyran-4-one, THPO) and N^1^–H (tris(6-methyl-3-hydroxypyridin-4-one), THP^H^) groups. The effect of these structural modifications on lipophilicity, gallium binding and metal ion selectivity was investigated. THP^H^ was able to bind ^68^Ga in extremely mild conditions (5 min, room temperature, pH 6, 1 μM ligand concentration) and, notably, *in vivo*, when administered to a mouse previously injected with ^68^Ga acetate. The ^67^Ga radiolabelled complex was stable in serum for more than 7 days. [^68^Ga(THP^H^)] displayed a log *P* value of –2.40 ± 0.02, less negative than the log *P* = –3.33 ± 0.02 measured for [^68^Ga(THP^Me^)], potentially due to an increase in intramolecular hydrogen bonding attributable to the N^1^–H pyridinone units. Spectrophotometric determination of the Ga^3+^/Fe^3+^ complex formation constants for both THP^Me^ and THP^H^ revealed their preference for binding Ga^3+^ over Fe^3+^, which enabled selective labelling with ^68^Ga^3+^ in the presence of a large excess of Fe^3+^ in both cases. Compared to THP^Me^, THP^H^ showed significantly reduced affinity for Fe^3+^, increased affinity for Ga^3+^ and improved radiolabelling efficiency. THPO was inferior to both THP^H^ and THP^Me^ in terms of labelling efficiency, but its benzylated precursor Bn-THPO (tris(6-methyl-3-benzyloxypyran-4-one)) provides a potential platform for the synthesis of a library of THP compounds with tunable chemical properties and metal preferences.

## Abstract 2

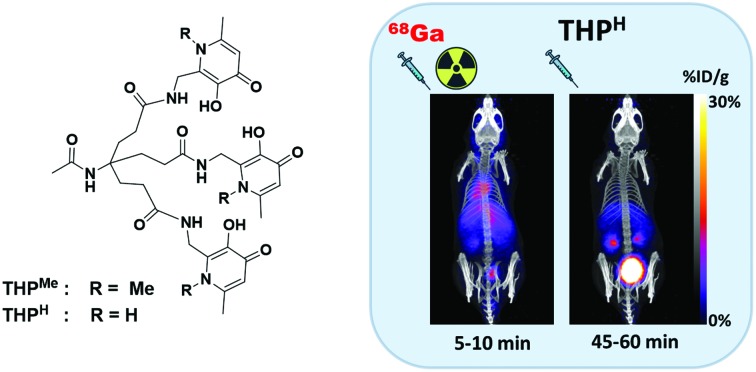
The outstanding efficiency of the tris(hydroxypyridonone) ligand THP^Me^ for radiolabelling PET radiotracers with ^68^Ga is surpassed by THP^H^.

## Introduction

Positron emission tomography (PET) is a non-invasive clinical diagnostic technique to visualise molecular processes *in vivo*. Gallium-68 (^68^Ga) has become a popular radionuclide for PET imaging, due to its favourable decay properties, generator-based availability and convenient half-life (68 min).^1^–^3^ Most ^68^Ga PET imaging exploits peptides labelled with ^68^Ga *via* an appropriate bifunctional chelator, to target specific disease-related receptors. ^68^Ga-DOTATATE, ^68^Ga-DOTATOC and ^68^Ga-DOTANOC, all of which contain peptides targeting the somatostatin receptor type II (SSTR2), have become clinical standards for imaging neuroendocrine tumours,^4^ while ^68^Ga-PSMA, containing a Glu-urea-Lys moiety targeting the prostate-specific membrane antigen (PSMA, glutamate carboxypeptidase II) shows great promise for prostate cancer imaging in clinical trials.^5^,^6^

An attractive attribute of ^68^Ga is that, in principle, bifunctional chelators that bind gallium rapidly and with high affinity could be used for single-step kit-based radiolabelling, minimising handling of radioactivity and avoiding difficult, time-consuming radiosynthesis and purification steps.^7^ For optimal efficiency and convenience at the point of use it is important to minimise reaction time, radiation dose to operators and dependency on costly automated synthesis equipment. Therefore, an ideal chelator for ^68^Ga must (i) bind Ga^3+^ in mild conditions, without need for pre-processing of the ^68^Ga generator eluate or purification of the radiolabelled complex; (ii) give complexes of high kinetic/thermodynamic stability that are resistant to *in vivo* transchelation; and (iii) produce a single well-defined radiolabelled species. Because the concentration of ^68^Ga^3+^ in generator eluates is very low, preference for Ga^3+^ over other metal contaminants in eluates or in equipment used for radiolabelling (vials, syringes *etc.*) is also important to obtain radiotracers with high molar activity.

Despite efforts to develop suitable chelators (Fig. S1†) for ^68^Ga radiolabelling, these ideals are only very recently being approached. The tetraazacyclododecane chelator DOTA, used in several ^68^Ga-peptide radiopharmaceuticals, requires harsh radiolabelling conditions (low pH, high temperature) not compatible with sensitive biomolecules, and long reaction times unsuitable for the 68 min half-life of ^68^Ga.^8^,^9^ The triazacyclononane chelator NOTA and its phosphinic acid derivatives of the TRAP family represent an advance on DOTA, often providing quantitative radiochemical yield (RCY) at room temperature and acidic pH.^8^,^10^,^11^ The “chimeric” DATA chelators, possessing both cyclic and an acyclic nitrogen atoms, can be radiolabelled quantitatively with ^68^Ga over a wide pH range and resist demetallation in the presence of transferrin or Fe^3+^.^12^,^13^ For all these chelators, radiolabelling with ^68^Ga is hampered particularly by the presence of Cu^2+^.^13^–^15^ Zn^2+^ also competes with Ga^3+^ for coordination to NOTA and DATA ligands.^15^,^16^ The acyclic chelator H_2_dedpa was radiolabelled with ^68^Ga at room temperature and acidic pH, achieving high molar activities.^17^

**Fig. 1:**
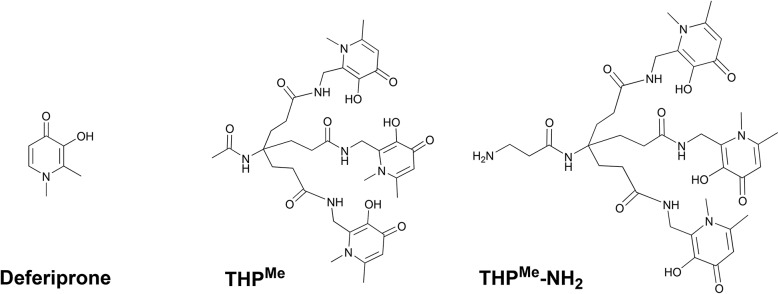
Hydroxypyridinone-based chelators. Left to right: Prototype bidentate ligand deferiprone; hexadentate ligand THP^Me^; THP^Me^-NH_2_, the basis of bifunctional derivatives.

Another class of Ga^3+^ ligands with potential to meet the above ideals is based on siderophores and iron chelators, exploiting the similarity between Ga^3+^ and Fe^3+^ in terms of charge, ionic radius (62 pm for Ga^3+^*vs.* 65 pm for high spin Fe^3+^ (ref. 18)) and preference for hard oxygen donors. The siderophore fusarinine-C (FSC),^19^ obtained from the fungus *Aspergillus fumigatus*, showed excellent ^68^Ga radiolabelling giving high molar activity over a wide (3–8) pH range. The bacterial siderophore deferoxamine (DFO-B) can also be radiolabelled with ^68^Ga in a wide pH range, but is subject to metal dissociation at low concentration^20^ and cannot compete effectively with other ^68^Ga chelators.^21^ The acyclic HBED (*N*,*N*-bis(2-hydroxybenzyl)ethylenediamine-*N*,*N*-diacetic acid), employed in a ^68^Ga PSMA tracer,^22^ binds gallium(iii) and iron(iii) with very high affinity (log *K*_Ga_ = 37.73, log *K*_Fe_ = 36.74 23). The tris(hydroxypyridinone) chelator THP^Me^ (Fig. 1, previously known as CP256 and THP^24^–^31^), investigated as a ^68^Ga chelator, was also initially developed as an iron-chelating agent.^32^ It has a tripodal scaffold supporting three pendant 3-hydroxypyridin-4-one (HP) units based on the bidentate chelating drug deferiprone (Fig. 1). Each arm can coordinate Ga^3+^ through the deprotonated hydroxyl and carbonyl groups.^33^ THP^Me^ is an efficient gallium chelator, out-competing other popular chelators^21^ and achieving quantitative radiolabelling in extremely mild conditions without eluate pre-processing or post-labelling purification,^24^ to produce a single [^68^Ga(THP^Me^)] species, unlike other chelators such as HBED.^21^ Bifunctional derivatives based on THP^Me^-amine conjugates (Fig. 1) have been used to produce several peptide and protein conjugates, with promising results. These include peptides targeting the SSTR2,^26^ α_v_β_3_ integrin,^27^ the prostate specific membrane antigen (PSMA)^28^ and small proteins.^34^ The PSMA-targeting THP^Me^ conjugate is now in phase 2 trials and routine clinical use in some centres.^30^,^35^–^37^ A dendrimer derivative of THP^Me^ (HP9) has also been developed to achieve higher molar activity ^68^Ga-bioconjugates.^29^

While ^68^Ga-THP^Me^-PSMA was comparable to its HBED counterpart in PET imaging of tumours,^28^ other derivatives such as the ^68^Ga-THP^Me^-TATE and ^68^Ga-THP^Me^-RGD_3_ demonstrated a lower tumour/non-target organ ratio compared to ^68^Ga radiotracers based on DOTA chelators,^27^,^31^ revealing how different targets may benefit from chelators with different chemical properties and, in turn, the potential value of structural variants on this promising platform. Another potential concern (albeit not arising in practice so far) is that similarity between Fe^3+^ and Ga^3+^ may lead to competition with adventitious Fe^3+^ (from vials, syringe needles radiolabelling equipment or generator eluate). Further optimisation of THP ligand design is therefore important. Modification at the ring nitrogen offers a straightforward way to tailor THP properties such as lipophilicity and hydrogen bonding capability. Previous studies on bidentate hydroxypyridinone ligands show that modification at the ring nitrogen is possible without significant detriment to their M^3+^ affinity.^33^,^38^

**Scheme 1:**
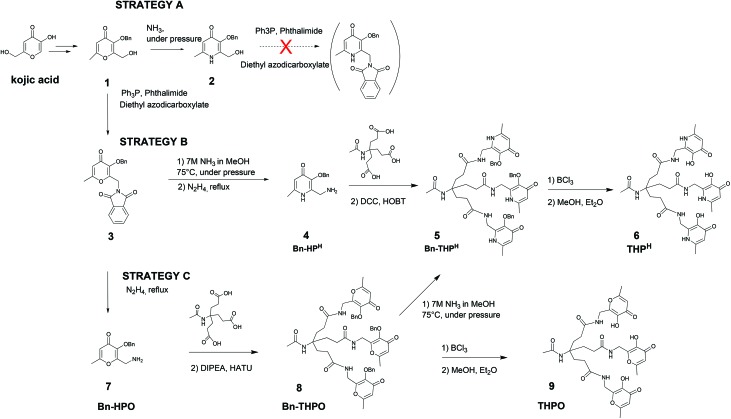
Synthetic pathways evaluated for synthesis of THP^H^ and its tris(hydroxypyranone) counterpart THPO.

Herein we present new synthetic routes to THP ligands, including the new derivative THP^H^ (Scheme 1) in which the *N*-methyl groups have been replaced by hydrogen. The labelling efficiency and lipophilicity of [^68^Ga(THP^H^)] is compared to its THP^Me^ and tris(hydroxypyranone) (THPO) analogues, and the conditional formation constants of the Ga^3+^ and Fe^3+^ complexes are evaluated, to measure and improve upon the gallium-selectivity and effectiveness of the prototype THP^Me^ for ^68^Ga radiolabelling.

## Results and discussion

### Synthesis

Synthesis of the precursor Bn-HP^H^ (**4**, Scheme 1) from **1** was first attempted using the previously reported approach to THP^Me^ synthesis^39^ (Scheme 1, **strategy A**), *i.e.*, conversion of **1** into pyridinone **2** by reaction with ammonia, followed by a Mitsunobu reaction with phthalimide and subsequent deprotection of the newly-introduced nitrogen with hydrazine at reflux. The Mitsunobu reaction failed to give the desired product, likely due to competition by the N^1^–H group with the phthalimide N–H group for deprotonation and subsequent S_N_2 reaction on the phosphonium-activated alcohol. New synthetic strategies were therefore developed.

In **strategy B**, the Mitsunobu reaction was performed directly on **1** (to avoid deleterious side reactions of the pyridinone N^1^–H group). The pyranone–pyridinone conversion was then carried out on **3**, followed by deprotection of the NH_2_ functionality to give the new primary amine-containing hydroxypyridinone, Bn-HP^H^ (**4**). This was coupled with the tripodal tricarboxylic acid^32^ to give Bn-THP^H^ (**5**) in 15.6% overall yield.

**Fig. 2:**
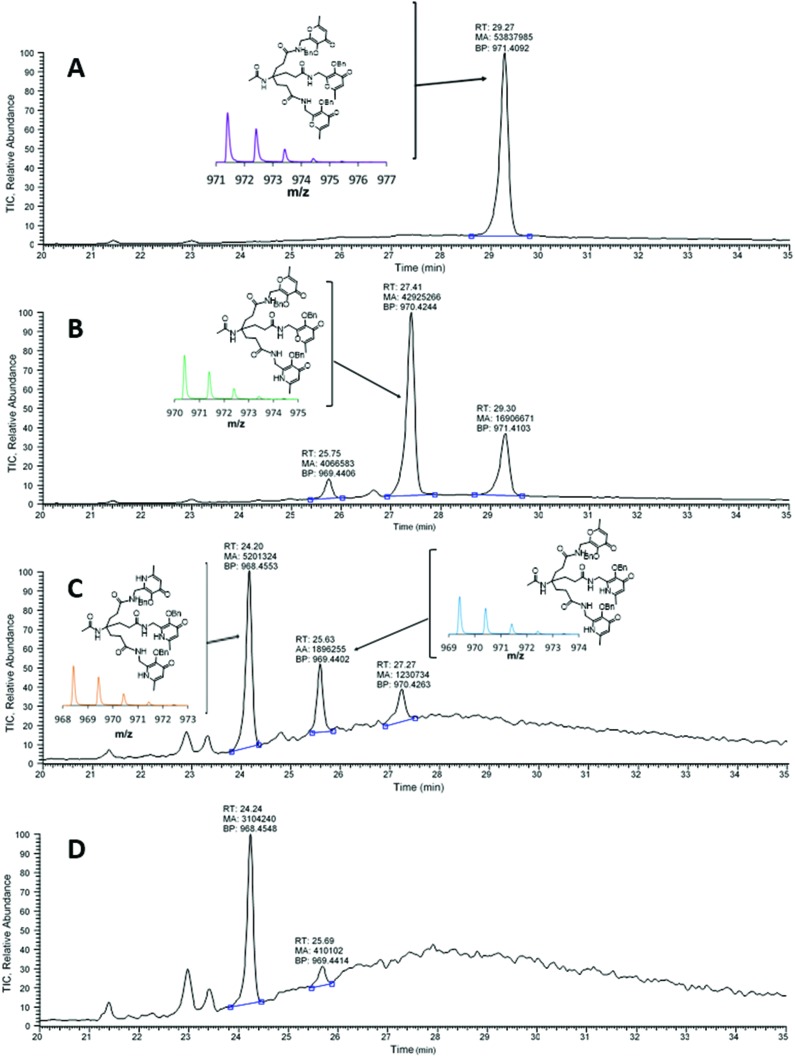
LC-MS analysis (total ion current) of the reaction mixture for conversion of Bn-THPO (**8**) to Bn-THP^H^ (**5**) (strategy C) at (A) 0 h (B) 8 h (C) 48 h and (D) 72 h. RT: Retention time; MA: area; BP: *m*/*z* value. Insets: Bn-THPO and MS signal of [Bn-THPO + H]^+^ (purple), mono-substituted Bn-THPO and MS signal (green), di-substituted Bn-THPO and MS signal (blue), Bn-THP^H^ structure and MS signal (orange).

In an alternative approach (**strategy C**), the pyranone–pyridinone conversion was performed after assembling the hexadentate tripodal unit. Compound **3** was deprotected to give the hydroxypyranone bearing a pendant primary amine, Bn-HPO (**7**), which was then coupled to the tricarboxylic acid to obtain the benzyl-protected tris(hydroxypyranone) Bn-THPO (**8**). Treatment of **8** with ammonia under pressure to give Bn-THP^H^ (**5**) was monitored using LC-MS (Fig. 2). The overall yield of Bn-THP^H^ (**5**) from compound **1***via***strategy C** (7.3%), was lower than that *via* strategy **B**, due to the additional step. However, in **strategy C** the benzylated tris(hydroxypyranone) precursor Bn-THPO holds great promise as a flexible platform for synthesising a versatile library of tris(hydroxypyridinone) compounds with varying substituents at the pyridyl nitrogen, by reaction with a large excess of the relevant primary amine. The LC-MS data show that the pyranone-to-pyridinone conversion proceeded in a step-wise fashion, offering the opportunity to create mixed species containing both pyranone and pyridinone units, or mixed pyridinone units.

Debenzylation of Bn-THP^H^ (**5**) with BCl_3_ produced the desired chelator THP^H^ (**6**) quantitatively. The same deprotection procedure applied to Bn-THPO (**8**) gave the tris(6-methyl-3-hydroxypyran-4-one) chelator THPO (**9**) in 78% yield.

### ^68^Ga radiolabelling and comparison with THP^Me^ and THPO

**Fig. 3:**
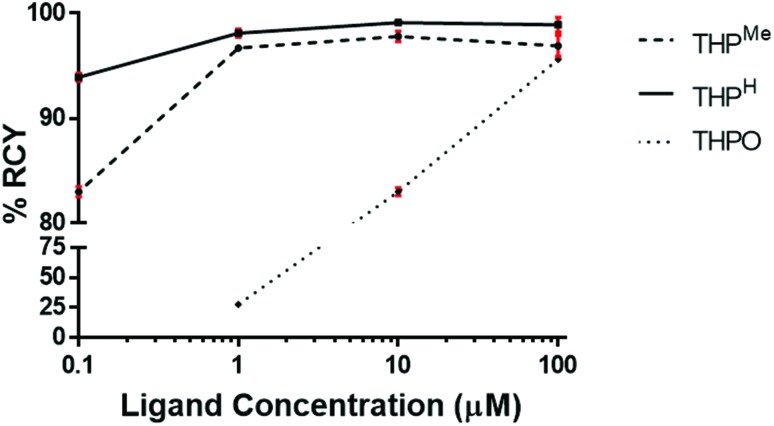
Comparison of RCY (average ± standard deviation, *N* = 3) obtained by ^68^Ga radiolabelling of THP^H^, THP^Me^ and THPO at decreasing ligand concentration.

THP^H^, THP^Me^ and THPO were radiolabelled at decreasing ligand concentrations in the same mild conditions (5 min, pH 6, room temperature) previously employed for THP^Me^.^24^ Radiochemical yields (RCY) are reported in Table 1 and compared graphically in Fig. 3.

**Table 1:** Log *P* and log *D*_7.4_ values and RCY, at different ligand concentrations, of ^68^Ga complexes (average ± standard deviation (*N* = 4 for log *P* and log *D*_7.4_, *N* = 3 for RCY)

	[Ga(THP^Me^)]	[Ga(THP^H^)]	[Ga(THPO)]
Log *P*	–3.33 ± 0.02	–2.40 ± 0.02	–1.64 ± 0.01
Log *D*_7.4_	–3.27 ± 0.02	–2.28 ± 0.05	–1.65 ± 0.03
**Radiochemical yield**			
100 μM	96.9 ± 1.0	98.9 ± 0.7	95.6 ± 0.2
10 μM	97.8 ± 0.5	99.1 ± 0.2	83.0 ± 0.4
1 μM	96.7 ± 0.2	98.1 ± 0.4	27.5 ± 0.7
0.1 μM^*a*^	83.0 ± 0.5	93.9 ± 0.4	N/A

^*a*^Radiolabelling performed with ^68^Ga eluate from a second E&Z generator, eluted with clinical grade HCl.

Efficient (>95%) radiolabelling of THP^H^ achieved at concentrations as low as 1 μM (Table 1), was verified by iTLC and HPLC analysis. At a ligand concentration as low as 0.1 μM THP^H^ was still able to bind ^68^Ga in 93.9% RCY while the RCY for THP^Me^ decreased to 83%, highlighting improved radiolabelling efficiency for the new chelator.

**Fig. 4:**
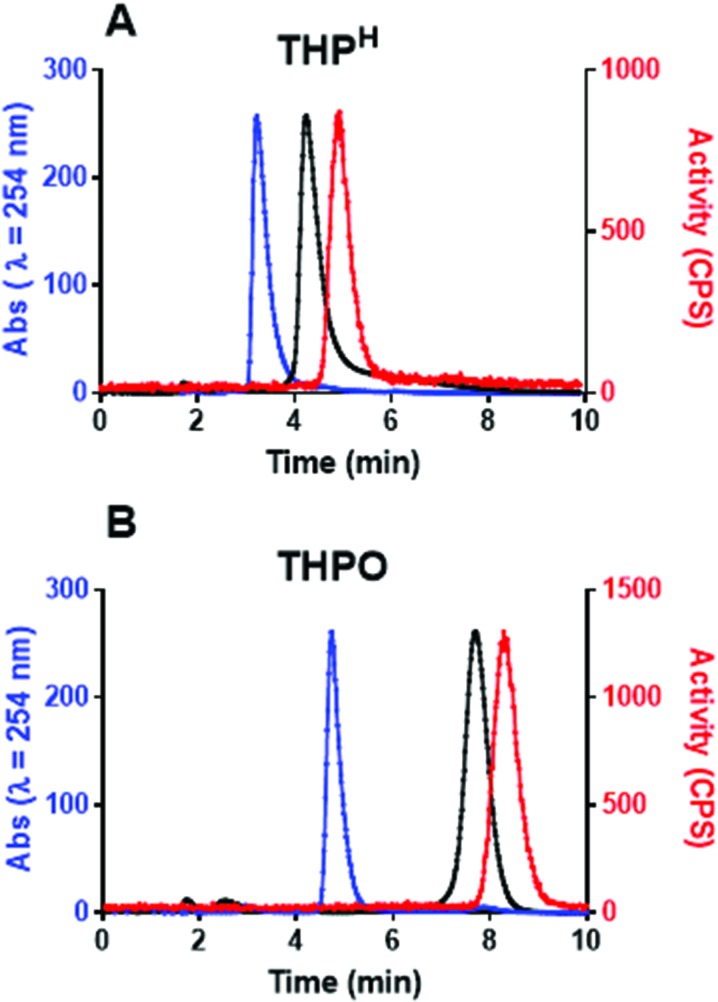
Normalised chromatograms of THP^H^ (A) and THPO (B) and their gallium complexes. Blue: UV (254 nm) for ligand; Black: UV (254 nm) for ^nat^Ga complex; Red: radiochromatogram for ^68^Ga complex. The serial configuration of the detectors accounts for a 36 seconds delay between UV and radio-chromatograms.

As was the case for [^68^Ga(THP^Me^)], no pre-processing of the generator eluate or post-labelling purification was required to obtain a radiochemically pure complex. iTLC showed only one species, corresponding to the radiolabelled complex without the presence of colloidal or unchelated “free” ^68^Ga. Analytical reversed-phase HPLC of the radiolabelling mixture (method 3) showed only one signal in the UV-Vis chromatogram (3 min 26 s), attributed to excess ligand, and one in the radiochromatogram at 4 min 56 s attributed to [^68^Ga(THP^H^)]. HPLC analysis of non-radioactive [^nat^Ga(THP^H^)] also showed a single peak in the UV-Vis chromatogram, whose retention time (4 min 20 s) matched that of [^68^Ga(THP^H^)] after correcting for delay due to the serial configuration of the detectors (Fig. 4A). The mass spectrum of [^nat^Ga(THP^H^)] confirmed the 1 : 1 stoichiometry of the complex (Fig. S4†), and no other gallium-containing ions were observed. As was the case for [Ga(THP^Me^)], low solubility of [^nat^Ga(THP^H^)] prevented further characterisation by ^1^H/^71^Ga NMR. Synthesis of more soluble conjugates of the ligands is underway to enable NMR studies.

The tris(hydroxypyranone) ligand THPO was also radiolabelled quantitatively, in the same conditions except that significantly higher ligand concentration (100 μM) was required to reach quantitative radiolabelling (Table 1). HPLC analysis (method 4) revealed a single peak at 8 min 18 s, matching the UV peak of [^nat^Ga(THPO)] at 7 min 42 s (Fig. 4B).

The observed decreased efficiency in gallium binding was not unexpected for pyranone derivatives, whose lower electron density of the heterocyclic ring compared to the pyridinone analogues is known to compromise binding to iron.^32^,^40^

Determination of the partition and distribution coefficients (log *P* and log *D*_7.4_, Table 1) for the ^68^Ga complexes of THP^Me^ and THP^H^ ligands revealed, unexpectedly, higher lipophilicity of [Ga(THP^H^)] than of [Ga(THP^Me^)], although both complexes were highly hydrophilic. This could be due to [Ga(THP^H^)] forming intramolecular hydrogen bonds rather than hydrogen bonds with the solvent. This phenomenon was previously reported for their bidentate analogues and some amido-3-hydroxypyridin-4-one ligands.^41^–^43^ As expected, [Ga(THPO)], in which oxygen replaces the heterocyclic amine, was more hydrophobic than its pyridinone counterparts. This agrees with the above LC-MS results for Bn-THPO and Bn-THP^H^, where sequential replacement of the 3 oxygen atoms with N^1^–H groups progressively reduced retention times.

### Serum stability and *in vivo* studies

The stability of a radiolabelled chelate in biological environments is critical to its utility in radiotracers. [^67^Ga(THP^H^)] stability in human serum was determined by size-exclusion HPLC, comparing the elution profile of the complex with that of unchelated ^67^Ga in serum. The longer half-life ^67^Ga was used instead of ^68^Ga, to allow more prolonged evaluation of stability. [^67^Ga(THP^H^)] was stable in serum for at least 8 days with no shift in its chromatographic signal (retention time: 15 min 30 s) compared to the complex incubated in PBS. When unchelated ^67^Ga was incubated in serum, by contrast, ^67^Ga eluted at 12 min at early serum incubation time points (chelated as ^67^Ga EDTA by EDTA in the mobile phase, Fig. S7†), but became more associated with serum proteins over time: after 8 days incubation two new signals appeared, at 12 min [^67^Ga(EDTA)] and 9 min 30 s (serum proteins). Excellent serum stability has been previously observed for [^67^Ga(THP^Me^)], which showed no sign of transchelation after 4 h at 37 °C.^24^

**Fig. 5:**
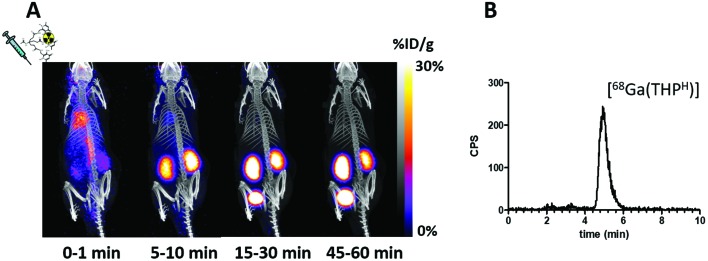
(A) Dynamic PET/CT MIP in a mouse injected with [^68^Ga(THP^H^)]. Fast blood clearance is evident, with only kidneys and bladder visible in the 15–30 min image. (B) radioHPLC of urine 60 min after injection (method 3), showing a single peak attributed to [^68^Ga(THP^H^)] (Fig. 4A).

A preliminary evaluation of the behaviour and stability of [^68^Ga(THP^H^)] *in vivo* was performed. PET imaging of a SCID beige mouse injected with [^68^Ga(THP^H^)] revealed rapid renal excretion (Fig. 5A). Reversed-phase HPLC of urine at 60 min post injection revealed a single radioactive species, corresponding to intact [^68^Ga(THP^H^)] (4.9 min, Fig. 5B). A similar biodistribution has been reported for [Ga(THP^Me^)].^24^

**Fig. 6:**
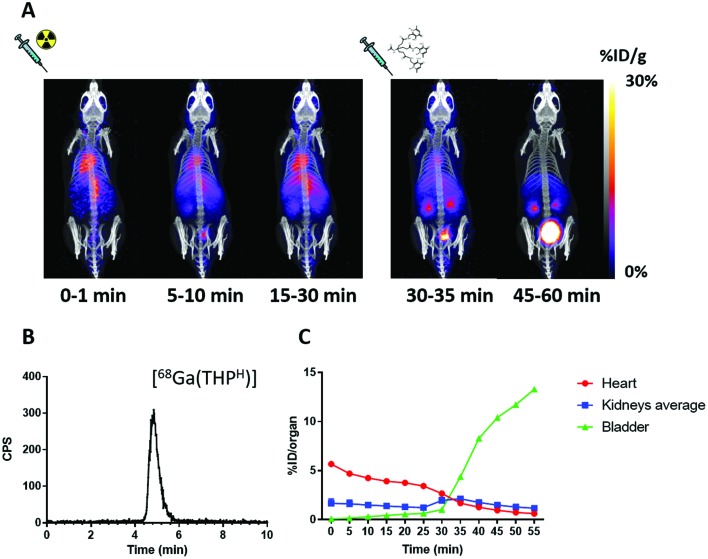
(A) Dynamic PET/CT MIP of a mouse injected with acetate buffered ^68^Ga at time 0 followed by THP^H^ at 30 min. Blood clearance of ^68^Ga acetate (represented by radioactivity in the heart ventricles) is slow in the first 30 min. After the injection of THP^H^ a sudden clearance occurs through the kidneys into the bladder. (B) RadioHPLC of urine 60 min after ^68^Ga injection (method 3), showing only one peak, attributed to [^68^Ga(THP^H^)]. (C) Time/activity curves showing % ID in heart (as a measure of blood activity), kidneys and bladder as a function of time. Each data point represents a 5 min interval defined by its starting time.

The ability of THP^H^ to scavenge gallium *in vivo* was also investigated. Fig. 6 shows how ^68^Ga biodistribution in a mouse injected with acetate-buffered ^68^Ga^3+^ suddenly changed upon injection of the chelator: most of the activity previously in the blood pool cleared quickly from the blood into the kidneys and the bladder (Fig. 6A). HPLC analysis of urine confirmed *in vivo* radiolabelling of the chelator (Fig. 6B), showing one peak corresponding to [^68^Ga(THP^H^)]. This scavenging ability, shared with THP^Me^ (ESI, Fig. S8†), reflects the rapid complexation kinetics and the extraordinary ability to transchelate gallium (which is known to bind rapidly and almost completely to transferrin when intravenously injected^44^) rapidly in the biological milieu.

### Spectrophotometric determination of conditional formation constants

The THP^Me^ and THP^H^ acid dissociation and conditional formation constants for their Ga^3+^ and Fe^3+^ complexes were investigated by spectrophotometric measurements. p*K*_a_ and pM values (defined as –log[M^3+^] when [ligand]_total_ = 10 μM, [metal]_total_ = 1 μM and pH = 7.4) are reported in Table 2. A full list of the measured conditional formation constants is provided in the ESI (Tables S1–S3†), together with speciation plots calculated for the pH range of the titration. The p*K*_a_ values for THP chelators are better described as intrinsic protonation constants since they represent the average p*K*_a_ values of the three hydroxypyridinone units, which could not be distinguished by UV spectrophotometric measurements.^45^ The first intrinsic p*K*_a_ is attributed to the carbonyl group of the HP unit, and the second to the adjacent hydroxyl group. For THP^H^ a third intrinsic protonation constant, p*K*_a3_, associated with deprotonation of the N^1^–H group in the pyridinone ring, exists outside the pH range used. The value of p*K*_a3_ was estimated to be 13 from the spectral change of the [Fe(THP^H^)] complex in the pH 10–12.5 range. The measured protonation constants for THP^H^ are lower than those measured for deferiprone, but slightly higher than those for THP^Me^ (that is, THP^Me^ is slightly more acidic than THP^H^). This was unexpected considering the presence of electron-donating *N*-methyl group and suggests that other chemical interactions (*e.g.* hydrogen bonding) may be influencing the p*K*_a_ of these compounds.

**Table 2:** Acid dissociation and conditional Fe^3+^ and Ga^3+^ complex formation constants for THP^Me^ and THP^H^. Estimated overall error for each value <3%. pM values were calculated based on [M]_total_ = 1 μM, [L]_total_ = 10 μM and pH = 7.4

	THP^Me^	THP^H^	Deferiprone^*a*^
p*K*_a1_	3.2^*b*^	3.4^*b*^	3.5
p*K*_a2_	9.4^*b*^	9.5^*b*^	9.8
p*K*_a3_	N/A	13.0^*c*^	N/A
pFe	29.1	28.6	20.8
pGa	30.0	31.8	20.7

^*a*^Previously reported measurements on deferiprone^33^ were repeated here to confirm the reliability of our titration method.

^*b*^Intrinsic p*K*_a_ values^45^ were determined from spectrophotometric titration. When deviation from the obtained intrinsic p*K*_a_ values were considered (±0.2–0.8 log units), no significant changes in pM values (less than 3%) were observed.

^*c*^Estimated p*K*_a_ value. No significant spectral change was observed between pH 11.5 and 12.5 for p*K*_a_ titration. Changing the estimated p*K*_a_ value can result in different metal log *β* values for THP^H^ but no appreciable change in pM values.

The data in Table 2 show that THP^H^ has higher affinity than THP^Me^ for Ga^3+^ but, unexpectedly, lower than THP^Me^ for Fe^3+^. The conditional formation constants indicate that both THP^H^ and THP^Me^ are selective for Ga^3+^ over Fe^3+^. This was unexpected considering that deferiprone lacks a preference for either metal,^33^ implying that the tripodal scaffold is critical in determining the coordination preferences of these hexadentate chelators.

Achievement of thermodynamic equilibrium for Ga^3+^ and Fe^3+^ complex formation/dissociation is a relatively slow process.^23^ Therefore, traditional spectrophotometric and potentiometric techniques, which allow only a few minutes equilibration, may produce apparent stability constants harbouring a kinetic component. In contrast, batch titrations, with equilibration over several days/weeks, generally result in more accurate “true” equilibrium stability constants. This discrepancy was recently acknowledged by Notni *et al.* for [Ga(TRAP-Pr)].^46^ In the case of THP chelators, no significant changes in the absorbance spectra were observed beyond the equilibration period, suggesting that equilibrium had been reached, although changes after longer intervals cannot be excluded. In any case, the use of a short equilibration time is particularly relevant for radiopharmaceutical applications, where radiolabelling processes must be rapid.

### Competition experiments

**Fig. 7:**
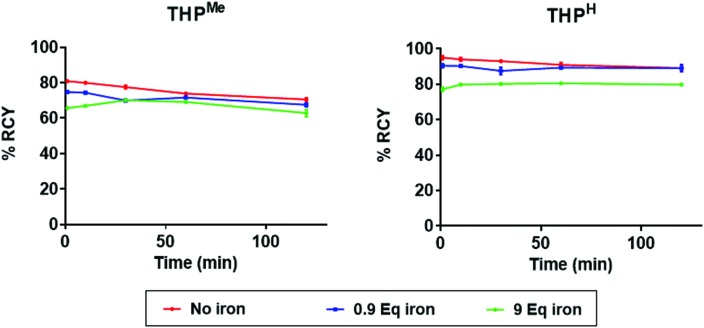
Influence of various concentrations of Fe^3+^ on the radiochemical yield of [^68^Ga(THP^Me^)] and [^68^Ga(THP^H^)] in the presence of 1 equivalent of ligand (10 μM) and 0.9 equivalent of ^nat^Ga^3+^ (average ± standard deviation; for control samples (without Fe) *N* = 6, for other samples *N* = 3 (error bars are too small to be visible).

To quantify the preference of the two ligands for Ga^3+^ over Fe^3+^ in a radiolabelling setting, a competition experiment was performed, where ^68^Ga radiolabelling of THP^Me^ and THP^H^ in the presence of different concentrations of Fe^3+^ was investigated. Instead of a traditional “no-carrier-added” radiolabelling mixture, in these competition studies non-radioactive ^nat^Ga^3+^ was added to ^68^Ga eluate (to reach a gallium : ligand ratio of approx. 9 : 10 to introduce competition by ensuring that the ligand concentration was sufficient to bind just one of the two metals quantitatively). Since ^68^Ga is chemically indistinguishable from natural gallium and its addition negligibly increases the total concentration of Ga^3+^, the percentage of ^68^Ga^3+^ bound to the ligand reflects the percentage of total Ga^3+^ bound to the ligand. To ensure constant pH at different metal concentrations, a higher concentration of ammonium acetate buffer than in conventional radiolabelling experiments was necessary (final concentration 0.44 M). Upon addition of the THP ligand (≈1 equivalent, 10 μM) to mixtures of ^nat/68^Ga^3+^ (0.9 equivalent) and Fe^3+^ (0, 0.9 or 9 equivalents) the radiochemical yield of the radiolabelling mixture was measured by iTLC (mobile phase 2) at different time points (Fig. 7).

Unexpectedly, under these conditions (unlike conventional radiolabelling conditions) THP^Me^ could not reach quantitative radiochemical yield even in the absence of iron, probably because the higher buffer and metal salt concentration increases the abundance of competing ligands (ammonia, acetate, nitrate) compared to more conventional labelling media. Nonetheless, it is clear that despite the presence of insufficient ligand to bind all the Ga^3+^ and Fe^3+^, the ^68^Ga-labelling efficiency was not dramatically reduced by the presence of even a ten-fold excess (compared to ligand) of Fe^3+^. Notably, the presence of 0.9 equivalents of iron with 0.9 of gallium decreased the radiochemical yield for the two ligands only marginally (not significantly for either ligand at later time points, *p* = 0.06 for THP^Me^, *p* = 0.22 for THP^H^), indicating that iron could not compete effectively with gallium for ligand binding. When the amount of iron was increased to 9 equivalents (a ten-fold excess over gallium), still the majority of the ligand bound to Ga^3+^ and not Fe^3+^, indicating a clear preference of THP ligands for gallium over iron (≈40 fold preference for gallium over iron under these conditions was estimated considering that a ≈80 : 20 gallium : iron complex ratio prevailed when a 10 fold excess of iron over gallium was present). This strong preference of both THP^Me^ and THP^H^ for binding Ga^3+^ over Fe^3+^ indicates that the presence of significant amounts of iron should not adversely affect the performance of the chelators during radiolabelling or *in vivo*.

**Fig. 8:**
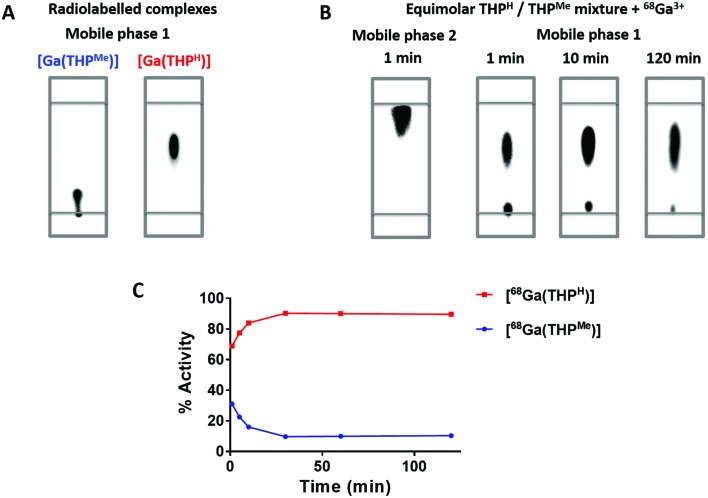
iTLCs of the radioactive ^68^Ga complexes of THP^Me^ and THP^H^ in mobile phase 1 (A) and of an equimolar mixture of THP^Me^ and THP^H^ treated with ^68^Ga^3+^ in mobile phase 2 (1 min time point) or in mobile phase 1 (1, 10 and 120 min) (B), imaged with a phosphorimager. (C) Shows how the percentage of activity associated with the two chelators, as calculated from the images, changes over time. Data are average ± standard deviation (*N* = 3, error bars are too small to be visible).

A second competition study between THP^Me^ and THP^H^ was conducted, to determine whether their different affinities for gallium would affect their relative ^68^Ga labelling efficiency. The difference in retention factor between the two complexes on iTLC (mobile phase 1) was exploited to monitor a solution in which ^68^Ga was added to an equimolar mixture of the two ligands (100 μM each, with equimolarity ensured by integration of the ^1^H NMR spectrum, Fig. S6†), by sampling at different times after the addition (Fig. 8). After one minute the radioactivity was already quantitatively chelated, indicating extremely fast radiolabelling for both compounds, but with preference for THP^H^ (ratio: 70 : 30). By 120 min the ratio had increased to ≈90 : 10, confirming that gallium binds preferentially to THP^H^ rather than THP^Me^. These data agree qualitatively with the spectrophotometric measurements and suggest that binding to both ligands is initially under kinetic control, while the thermodynamic preference for THP^H^ is established by 30 min. Interestingly, these results also demonstrate that Ga^3+^ can transchelate from THP^Me^ to THP^H^ under these conditions. While this suggests a degree of kinetic lability of the [Ga(THP)] system when excess ligand is present, resistance towards transchelation *in vivo* (where excess ligand is greatly diluted) has been extensively confirmed in previous preclinical and clinical studies,^24^,^26^–^30^ including the present manuscript (Fig. 5).

Both spectrophotometric titration and competition experiments show how modification at the hydroxypyridinone ring unexpectedly influences the preference of THP chelators for Ga^3+^*vs.* Fe^3+^, whereas a similar effect was not observed for bidentate hydroxypyridinone compounds.^38^ This implies that geometric restrictions imposed by the tripodal structure of the ligand are critical to metal ion selectivity. It is likely that replacing the *N*^1^-methyl group with hydrogen modifies the steric constraints and the degree and type (*e.g.* intramolecular *vs.* intermolecular) of hydrogen bonding, compared to THP^Me^. Both these factors could result in marked changes in the geometry and rigidity of the coordination sphere, leading to different metal affinities and selectivity of the two THP chelators.

## Conclusions

Despite the acyclic topology of THP^Me^ and THP^H^, their affinity for Ga^3+^ is very high (pGa = 30.0 and 31.8 respectively) and, contrary to our naive expectation, exceeds their affinity for Fe^3+^. The reasons for this preference are unknown, but likely related to geometry constraints imposed by the tripodal framework, since no such preference was observed for the bidentate chelator deferiprone.^33^ An important consequence of this selectivity is that the expected vulnerability of the THP ligands to competition with iron, during labelling and *in vivo*, is not manifested either in the measurements described here or in the preclinical and clinical uses of THP^Me^ described so far.^24^,^26^–^30^,^35^–^37^ Moreover, minor alteration of the hydroxypyridinone moiety (replacing N^1^–CH_3_ with N^1^–H) significantly improves both the affinity for Ga^3+^ (and hence ^68^Ga radiolabelling efficiency) and the selectivity for Ga^3+^ over Fe^3+^ (and hence resistance to interference from adventitious iron). The origin of this effect may be related to changes in intramolecular hydrogen bonding, which affect the ability of the scaffold to provide an idealised octahedral cavity of appropriate size, for as yet unknown reasons. Synthesis of more soluble derivatives is underway to allow investigation of these factors by NMR. As well as producing THP^H^ as a new tris(hydroxypyridinone) ligand with superior Ga^3+^-chelating properties compared to the established THP^Me^, we have described a novel synthetic strategy that allows further systematic modification of pyridinone nitrogen substituents by using the protected THPO as a common precursor. This opens the door to further tuning of ligand design to improve labelling efficiency and modify biological behaviour.

## Experimental section

### Materials and instrumentation

Chemicals were obtained from Sigma Aldrich, unless otherwise specified, and used without further purification. NMR spectra were acquired on a Bruker Advance 400 spectrometer with a 5 mm Quattro Nucleus Probe (QNP) at 400.13 MHz. Chemical shifts are referenced to the appropriate solvent peak. Positive ion mass spectra were recorded using an Agilent 6510 QTOF spectrometer. Analytical reversed-phase LC-MS were acquired on a Thermo Scientific Exactive Orbitrap Mass Spectrometer coupled to a Thermo Scientific Accela Pump with CTC Autosampler, using a ThermoFisher HyperSil GOLD column (2.1 × 150 mm, 5 μm), flow rate 0.2 mL min^–1^. Data were acquired and reference mass-corrected *via* a dual-spray electrospray ionisation source, using the factory-defined calibration procedure. Semi-preparative HPLC was carried out using an Agilent Eclipse XDB-C18 column (9.4 × 250 mm, 5 μm), flow rate 3 mL min^–1^, and UV detection at 214 nm on an Agilent 1200 LC system. Mobile phase A was water with 0.1% TFA and mobile phase B was acetonitrile with 0.1% TFA. For method 1, concentration of B increased from 0 to 100% at 1% min^–1^. For method 2, concentration of B increased from 20% to 100% in 100 min. Analytical reversed-phase HPLC was performed on the same system using an Agilent Eclipse XDB-C18 column (4.6 × 150 mm, 5 μm) with a 1 mL min^–1^ flow rate and UV detection at 214 or 254 nm, coupled to a LabLogic Flow-Count radioactivity detector with a sodium iodide probe (B-FC-3200). Mobile phase A was water with 0.1% TFA, mobile phase B was acetonitrile with 0.1% TFA. For methods 3 and 4, UV detection was set at 254 nm and isocratic elution was used with 10% B and 15% B, respectively. For methods 5 and 6, UV detection was set at 214 nm and gradients included 5 min of equilibration at 0% B at the start of the run. For method 5, concentration of B increased from 0% (at 5 min) to 45% (at 15 min) and subsequently decreased to 0 (at 20 min). For method 6, % B increased from 0% (at 5 min) to 75% (15 min) and back to 0% (20 min). Size-exclusion chromatography was conducted using a BioSep SEC-s2000 column (145 Å, 300 × 7.8 mm, 5 μm) with a mobile phase of PBS with 50 mM EDTA trisodium salt, flow rate 1 mL min^–1^. ^68^Ga was obtained from an Eckert & Ziegler ^68^Ge/^68^Ga-generator eluted with high-purity 0.1 M HCl (Fluka Analytical). Instant thin layer chromatography strips (iTLC-SG, Varian Medical Systems) were run in two different mobile phases (mobile phase 1: 0.1 M citrate pH 5; mobile phase 2: 1 : 1 ammonium acetate 2 M : methanol) and visualised using a Cyclone Plus Phosphor Imager (PerkinElmer) and a Raytest Rita-Star TLC scanner.

### Synthesis and characterisation data

2-Hydroxymethyl-3-benzyloxy-6-methyl-pyran-4(1*H*)-one (**1**) was synthesised following published procedures.^39^ THP^H^ was synthesised from **1** employing two different synthetic strategies using reactions based on literature methods.^32^,^39^ The tripodal scaffold 4-acetamido-4-(2-carboxyethyl)heptanedioic acid was synthesised following literature procedures.^32^ All intermediates and final products were characterised by ESI-MS, ^1^H NMR and ^13^C NMR (Fig. S9–S16†).

### 2-Hydroxymethyl-3-benzyloxy-6-methyl-pyridin-4(1*H*)-one (**2**)

A solution of **1** (1.010 g, 4 mmol) in ethanol (5 mL) was sealed in a thick-walled glass vial containing 25% aqueous ammonia (20 mL) and stirred at 75 °C overnight. Addition of concentrated HCl to reach neutral pH precipitated white crystals, which were collected by filtration and washed with cold water and diethyl ether (0.609 g, 60.6%). **ESI-MS (*m*/*z*):** 246.12 [M + H]^+^ calcd: 246.11 for C_14_H_15_NO_3_ + H^+^. ^**1**^**H NMR** (methanol-*d*_4_, 400 MHz) *δ*: 2.33 (s, 3H, C^6^–**CH**_**3**_), 4.34 (d, 2H, C^2^–**CH**_**2**_–OH), 5.08 (s, 2H, C^3^–O–**CH**_**2**_–Ph), 6.33 (s, 1H, **C**^**5**^**–H** in pyridinone), 7.35 (m, 5H, C^3^–O–CH_2_–**Ph**). ^**13**^**C NMR** (methanol-*d*_4_, 100 MHz) *δ*: 18.7 (C^6^–**CH**_**3**_), 57.0 (C^2^–**CH**_**2**_–OH), 74.6 (C^3^–O–**CH**_**2**_–Ph) 117.1 (**C**^5^**–H** in pyridinone), 128.0 (*o*-**CH** in benzyl), 129.4 (*p*-**CH** in benzyl), 130.2 (*m*-**CH** in benzyl), 138.6 (i-**C**–CH_2_ in benzyl), 143.0 (**C**^2^ in pyridinone), 144.7 (**C**^3^ in pyridinone), 147.8 (**C**^6^ in pyridinone), 176.0 (**C**^4^ in pyridinone).

### 2-Phthalimidomethyl-3-benzyloxy-6-methyl-pyran-4(1*H*)-one (**3**)

A solution of **1** (1.05 g, 4.0 mmol), triphenylphosphine (1.395 g, 8 mmol) and phthalimide (1.181 g, 8 mmol) in dry tetrahydrofuran (20 mL) under an atmosphere of nitrogen was cooled to 0 °C and diethyl azodicarboxylate (2 mL, 8 mmol) was added dropwise. After stirring overnight, methanol was added to quench excess diethyl azodicarboxylate prior to solvent removal by rotary evaporation. The white residue was recrystallised from methanol affording white crystals (1.157 g, 72.3%). **ESI-MS (*m*/*z*):** 376.11 [M + H]^+^, calcd: 376.12 for C_22_H_17_NO_5_ + H^+^. ^**1**^**H NMR** (DMSO-*d*^6^, 400 MHz) *δ*: 2.14 (s, 3H, C^6^–**CH**_**3**_), 4.73 (s, 2H, C^2^–**CH**_**2**_–N), 5.10 (s, 2H, C^3^–O–**CH**_**2**_–Ph), 6.26 (s, 1H, **C**^**5**^**–H** in pyranone), 7.40 (m, 5H, C^3^–O–CH_2_–**Ph**), 7.89 (m, 4H, phthalimide). ^**13**^**C NMR** (DMSO-*d*_6_, 100 MHz) *δ*: 19.0 (C^6^–**CH**_**3**_), 34.4 (C^2^–**CH**_**2**_–N), 72.9 (C^3^–O–**CH**_**2**_–Ph), 114.3 (**C**^5^**–H** in pyranone), 123.4 (**C**^**7**^**–H** and **C**^4^**–H** in phthalimide), 128.2 (*o*-**CH** in benzyl), 128.4 (*p*-**CH** in benzyl), 128.5 (*m*-**CH** in benzyl), 131.4 (**C**^**5**^**–H** and **C**^**6**^**–H** in phthalimide), 134.7 (**C**^**3a**^ and **C**^**7a**^ in phthalimide), 136.8 (i-**C**–CH_2_ in benzyl), 142.6 (**C**^**2**^ in pyranone), 153.9 (**C**^**3**^ in pyranone), 165.1 (**C**^6^ in pyranone) 167.2 (**C**=O in phthalimide), 174.6 (**C**^**4**^ pyranone).

### Bn-HP^H^ (2-aminomethyl-3-benzyloxy-6-methyl-pyridin-4(1*H*)-one) (**4**)

A solution of **3** (500 mg, 1.3 mmol) in 7 M ammonia solution in methanol (30 mL) was added to a thick-walled glass vial that was sealed and stirred overnight at 75 °C. Aqueous hydrazine (55%, 0.2 mL) was then added to the reaction mixture and the solution heated at reflux for 3 h. After removal of the solvent by rotary evaporation the brown residue was purified by silica-gel chromatography (CH_2_Cl_2_ : MeOH = 80: 20, *R*_f_ = 0.35) to afford Bn-HP^H^ (317 mg, 45%).

**ESI-MS (*m*/*z*):** 245.13 [M + H]^+^, calcd: 245.13 for C_14_H_16_N_2_O_2_ + H^+^. ^**1**^**H NMR** (methanol-*d*_4_, 400 MHz) *δ*: 2.33 (s, 3H, C^6^–**CH**_**3**_), 3.60 (s, 2H, C^2^–**CH**_**2**_–NH_2_), 5.14 (s, 2H, C^3^–O–**CH**_**2**_–Ph), 6.39 (s, 1H, **C**^**5**^**–H** in pyridinone), 7.36 (m, 5H, C^3^–O–CH_2_–**Ph**). ^**13**^**C NMR** (methanol-*d*_4_, 100 MHz) *δ*: 19.4 (C^6^–**CH**_**3**_), 38.8 (C^2^–**CH**_**2**_–NH_2_), 74.6 (C^3^–O–**CH**_**2**_–Ph) 116.7 (**C**^5^**–H** in pyridinone), 129.6 (*o*-**CH** in benzyl), 129.6 (*p*-**CH** in benzyl), 130.4 (*m*-**CH** in benzyl), 138.5 (i-**C**–CH_2_ in benzyl) 143.6 (**C**^**2**^ in pyridinone), 143.8 (**C**^**3**^ in pyridinone), 149.0 (**C**^6^ in pyridinone), 174.2 (**C**^**4**^ in pyridinone).

### Bn-THP^H^ (**5**) (from Bn-HP^H^)

4-Acetamido-4-(2-carboxyethyl)heptanedioic acid (20 mg, 0.07 mmol), hydroxybenzotriazole hydrate (HOBT, 32.7 mg, 0.21 mmol) and dicyclohexylcarbodiimide (DCC, 44 mg, 0.21 mmol) were dissolved in the minimum amount of *N*,*N*-dimethylformamide (DMF, ≈1.5 mL) and stirred. Bn-HP^H^ (86 mg, 0.35 mmol) was dissolved separately in 500 μL of DMF and added to the former mixture, which was then stirred at 50 °C for 48 h. Formation of the product over time was monitored *via* LC-MS (*m*/*z* = 968, [M + H]^+^). DMF was removed under high vacuum and the product purified by silica gel chromatography (eluent CH_3_OH : CHCl_3_ = 20 : 80 *R*_f_ = 0.25, followed by CH_3_OH : CHCl_3_ : 40% aqueous NH_3_ = 20 : 80 : 2) to obtain Bn-THP^H^ (37 mg, 48%). **ESI-MS (*m*/*z*):** 968.45 [M + H]^+^, 990.44 [M + Na]^+^, calcd: 968.46 for C_54_H_61_N_7_O_10_ + H^+^. ^**1**^**H NMR** (methanol-*d*_4_, 400 MHz) *δ*: 1.86 (s, 3H, **CH**_**3**_–CO–NH-tripod), 1.88 (m, 6H, CH_2_–**CH**_**2**_–CO–NH–CH_2_-pyridinone), 2.09 (m, 6H, **CH**_**2**_–CH_2_–CO–NH–CH_2_-pyridinone), 2.28 (s, 9H, C^6^–**CH**_**3**_), 4.10 (s, 6H, CO–NH–**CH**_**2**_-pyridinone), 5.11 (s, 6H, C^3^–O–**CH**_**2**_–Ph), 6.31 (s, 3H, **C**^**5**^**–H** in pyridinone), 7.35 (m, 15H, C^3^–O–CH_2_–**Ph**). ^**13**^**C NMR** (methanol-*d*_4_, 100 MHz) *δ*: 18.8 (C^6^–**CH**_**3**_), 23.6 (**CH**_**3**_–CO–NH-tripod), 30.8 (CH_2_–**CH**_**2**_–CO–NH–CH_2_-pyridinone), 31.0 (**CH**_**2**_–CH_2_–CO–NH–CH_2_-pyridinone), 37.7 (CH_2_–CH_2_–CO–NH–**CH**_**2**_-pyridinone), 58.9 (NH**C-**tripod), 74.5 (C^3^–O–**CH**_**2**_–Ph) 117.3 (**C**^**5**^**–H** in pyridinone), 129.5 (*p*-**CH** in benzyl), 130.2 (*m*-**CH** in benzyl), 138.5 (i-**C**–CH_2_ in benzyl), 141.4 (**C**^**2**^ in pyridinone), 144.8 (**C**^**3**^ in pyridinone), 148.0 (**C**^**6**^ in pyridinone), 173.0 (**C**^4^ in pyridinone), 176.1 (CH_2_–CH_2_–**CO–**NH–CH_2_-pyridinone).

### THP^H^ (**6**)

Bn-THP^H^ (17 mg, 0.018 mmol) was dissolved in the minimum amount of methanol (≈100 μL) and diluted to 1 mL with dichloromethane. An excess of BCl_3_ (3 mL of a 1 M solution in dichloromethane) was added through a cannula (Cole-Parmer) under an atmosphere of N_2_. After 2 hours, the vial was placed on ice and excess methanol was added to quench remaining BCl_3_. Volatiles were removed by rotary evaporation. The residue was redissolved in methanol and precipitated by addition of cold diethyl ether to give THP^H^ (3·HCl salt) (15.6 mg, 90% yield). **ESI-MS (*m*/*z*):** 698.32 [M + H]^+^, 349.67 [M + 2H]^2+^, 233.44 [M + 3H]^3+^, calcd: 698.31 for C_33_H_43_N_7_O_10_ + H^+^. ^**1**^**H NMR** (D_2_O, 400 MHz) *δ*: 1.85 (s, 3H, **CH**_**3**_–CO–NH-tripod), 1.87 (m, 6H, CH_2_–**CH**_**2**_–CO–NH–CH_2_-pyridinone), 2.17 (m, 6H, **CH**_**2**_–CH_2_–CO–NH–CH_2_-pyridinone), 2.47 (s, 9H, C^6^–**CH**_**3**_), 4.44 (d, 6H, CO–NH–**CH**_**2**_-pyridinone), 6.93 (s, 3H, **C**^**5**^**–H** in pyridinone). ^**13**^**C NMR** (D_2_O, 100 MHz) *δ*: 17.9 (C^6^-**CH**_**3**_), 22.6 (**CH**_**3**_–CO–NH-tripod), 28.9 (CH_2_–**CH**_**2**_–CO–NH–CH_2_-pyridinone), 29.2 (**CH**_**2**_–CH_2_–CO–NH–CH_2_-pyridinone), 36.6 (CH_2_–CH_2_–CO–NH–**CH**_**2**_-pyridinone), 58.0 (NH**C-**tripod), 111.7 (**C**^**5**^**–H** in pyridinone), 136.4 (**C**^**2**^ in pyridinone), 140.6 (**C**^**3**^ in pyridinone), 146.7 (**C**^**6**^ in pyridinone), 160.9 (**C**^4^ in pyridinone), 173.3 (CH_3_–**CO**–NH-tripod), 176.4 (CH_2_–CH_2_–**CO–**NH–CH_2_-pyridinone). **HPLC:** a single peak was detected using both HPLC methods 3 (*λ* = 254 nm, 3 min 26 s, Fig. 4A) and 5 (*λ* = 214 nm, 10 min 45 s, Fig. S2†).

### Bn-HPO (2-(aminomethyl)-3-(benzyloxy)-6-methyl-4*H*-pyran-4-one) (**7**)

A solution of **3** (499 mg, 1.33 mmol) was dissolved in ethanol (5 mL) and heated at reflux for 3 h after addition of 5.5% of aqueous hydrazine (1.5 mL). The pH was adjusted to 1 with concentrated HCl (37%) and the flask cooled to 0 °C. The phthalhydrazide precipitate was filtered out and the filtrate was evaporated to dryness. The resulting residue was dissolved in water (2 mL) and the pH was adjusted to 10 using concentrated NaOH (10 M). The solution was extracted with dichloromethane (4 × 2 mL), the organic layers were combined and the solvent removed by rotary evaporation. The crude product was purified by silica gel chromatography (dry loading, CH_2_Cl_2_ : MeOH = 90 : 10, *R*_f_ = 0.45) to obtain Bn-HPO (90.0 mg, 28% yield). **ESI-MS (*m*/*z*):** 246.11 [M + H]^+^, calcd: 246.11 for C_14_H_15_NO_3_ + H^+^. ^**1**^**H NMR** (methanol-*d*_4_, 400 MHz) *δ*: 2.31 (s, 3H, C^6^–**CH**_**3**_), 3.51 (s, 2H, C^2^–**CH**_**2**_–NH_2_), 5.09 (s, 2H, C^3^–O–**CH**_**2**_–Ph), 6.27 (s, 1H, **C**^**5**^**–H** in pyranone), 7.37 (m, 5H, C^3^–O–CH_2_–**Ph**). ^**13**^**C NMR** (methanol-*d*_4_, 400 MHz) *δ*: 19.5 (C^6^–**CH**_**3**_), 39.3(C^2^–**CH**_**2**_–NH_2_), 74.9 (C^3^–O–**CH**_**2**_–Ph) 115.0 (**C**^5^**–H** in pyridinone), 129.6 (*o*-**CH** in benzyl), 129.7 (*p*-**CH** in benzyl), 130.4 (*m*-**CH** in benzyl), 137.9 (i-**C**–CH_2_ in benzyl) 142.3 (**C**^**2**^ in pyranone), 162.9 (**C**^**3**^ in pyranone), 167.9 (**C**^**6**^ in pyranone), 178.4 (**C**^**4**^ in pyranone).

### Bn-THPO (**8**)

The tripodal acid 4-acetamido-4-(2-carboxyethyl)heptanedioic acid (14 mg, 0.049 mmol), diisopropylethylamine (25.6 μL, 0.15 mmol) and HATU (55.7 mg, 0.15 mmol) were dissolved in the minimum volume of *N*,*N*-dimethylacetamide (DMA, ≈500 μL), combined while stirring and left at room temp. for 1 h. A solution of Bn-HPO (52 mg, 0.16 mmol) in DMA (500 μL) was then added and the mixture stirred for 72 h. The DMA was removed under high vacuum and the product was purified using preparative HPLC (method 1) to obtain **Bn-THPO** (34.7 mg, 73.1% yield). **ESI-MS (*m*/*z*):** 486.21 [M + 2H]^2+^, 971.41 [M + H]^+^, 993.38 [M + Na]^+^, calcd: 971.41 for C_54_H_58_N_4_O_13_ + H^+^. ^**1**^**H NMR** (methanol-*d*_4_, 400 MHz) *δ*: 1.91 (s, 3H, **CH**_**3**_–CONH-tripod), 1.96 (m, 6H, CH_2_–**CH**_**2**_–CONH–CH_2_-pyranone), 2.17 (m, 6H, **CH**_**2**_–CH_2_–CONH–CH_2_-pyranone), 2.29 (s, 9H, C^6^–**CH**_**3**_), 4.23 (s, 6H, CONH–**CH**_**2**_-pyranone), 5.12 (s, 6H, C^3^–O–**CH**_**2**_–Ph), 6.28 (s broad, 3H, **C**^**5**^**–H** in pyranone), 7.35 (m, 15H, C^3^–O–CH_2_–**Ph**). ^**13**^**C NMR** (methanol-*d*_4_, 100 MHz) *δ*: 19.5 (C^6^–**CH**_**3**_), 23.5 (**CH**_**3**_–CO–NH-tripod), 30.9 (CH_2_–**CH**_**2**_–CO–NH–CH_2_-pyranone), 31.2 (**CH**_**2**_–CH_2_–CO–NH–CH_2_-pyranone), 37.3 (CH_2_–CH_2_–CO–NH–**CH**_**2**_-pyranone), 58.9 (NH**C-**tripod), 75.0 (C^3^–O–**CH**_**2**_–Ph) 115.2 (**C**^**5**^**–H** in pyridinone), 129.6 (*p*-**CH** in benzyl), 130.2 (*m*-**CH** in benzyl), 138.1 (i-**C**–CH_2_ in benzyl), 143.7 (**C**^2^ in pyranone), 159.1 (**C**^3^ in pyranone), 167.9 (**C**^6^ in pyranone), 172.9 (**C**^4^ in pyranone), 175.7 (CH_3_**–CO** 169.4), 178.2 (CH_2_–CH_2_–**CO–**NH–CH_2_-pyridinone).

### Bn-THP^H^ (**5**) (from Bn-THPO)

Bn-THPO (22.4 mg, 0.023 mmol) was added to 7 M ammonia in MeOH (11 mL) and the mixture stirred at 75 °C in a sealed thick glass vial. The reaction was left for 72 h and monitored *via* LC-MS (mobile phase: A = H_2_O + 0.1% formic acid, B = acetonitrile + 0.1% formic acid; gradient: 0–5 min 100% A, 5–55 min from 100% A to 100% B, flow rate 0.2 mL min^–1^, mass range 900–1100 *m*/*z*). The solvent was then removed under reduced pressure and the residue purified by preparative HPLC (method 2) to give Bn-THP^H^ (10.88 mg, 49% yield).

### THPO (**9**)

To Bn-THPO (6.65 mg, 0.007 mmol) in DCM : MeOH (7 : 1), BCl_3_ (3 mL of a 1 M solution in dichloromethane) was added *via* a cannula under a N_2_ atmosphere. After 2 h, the vial was placed on ice, the reaction was quenched with MeOH and the solvent was removed under reduced pressure. The residue was dissolved in water : acetonitrile (60 : 40) and purified by preparative HPLC (method 1) to give THPO (TFA salt, 5.6 mg, 78% yield). **ESI-MS (*m*/*z*):** 702.26 [M + H]^+^, 723.24 [M + Na]^+^, 351.13 [M + 2H]^2+^, calcd: 701.27 for C_33_H_41_N_4_O_13_ + H^+^. ^**1**^**H NMR** (methanol-*d*_4_, 400 MHz) *δ*: 1.91 (s, 3H, **CH**_**3**_–CO–NH-tripod), 1.99 (t, 6H, CH_2_–**CH**_**2**_–CO–NH–CH_2_-pyranone), 2.22 (t, 6H, **CH**_**2**_–CH_2_–CO–NH–CH_2_-pyranone), 2.30 (s, 9H, C^6^–**CH**_**3**_), 4.40 (d, 6H, CO–NH–**CH**_**2**_-pyranone), 6.24 (s, 3H, **C**^**5**^**–H** in pyranone). ^**13**^**C NMR** (methanol-*d*_4_, 100 MHz) *δ*: 19.7 (C^6^–**CH**_**3**_), 23.5 (**CH**_**3**_–CO–NH-tripod), 31.0 (CH_2_–**CH**_**2**_–CO–NH–CH_2_-pyranone), 31.3 (**CH**_**2**_–CH_2_–CO–NH–CH_2_-pyranone), 37.3 (CH_2_–CH_2_–CO–NH–**CH**_**2**_-pyranone), 59.0 (NH**C-**tripod), 112.3 (**C**^**5**^**–H** in pyranone), 143.5 (**C**^**2**^ in pyranone), 148.9 (**C**^**3**^ in pyranone), 167.5 (**C**^**6**^ in pyranone), 173.0 (**C**^**4**^ in pyranone), 176.1 (CH_3_–**CO**–NH-tripod), 176.4 (CH_2_–CH_2_–**CO–**NH–CH_2_-pyranone). **HPLC:** A single peak was observed with both HPLC methods 4 (*λ* = 254 nm, retention time 4 min 43 s, Fig. 4B) and 6 (*λ* = 214 nm, 11 min 10 s, Fig. S3†).

### Complexation with ^68^Ga^3+^ and ^nat^Ga^3+^

For all the radiolabelling experiments, an Eckert & Ziegler generator was eluted with 5 mL of high purity HCl 0.1 M (Fluka analytical) in five 1 mL fractions, whose activity was measured by a Capintec radionuclide dose calibrator. 100 μL of the highest activity fraction (15–20 MBq) were added to 100 μL of the ligand (concentration range 200–2 μM) in ammonium acetate 0.5 M. Verification of the radiolabelling was carried out after 5 min by reversed-phase HPLC (for THP^H^: method 3; for THPO: method 4) and iTLC-SG with two different mobile phases, as described above (mobile phase 1: *R*_f_ [Ga(THP^H^)] = 0.64 ± 0.02, *R*_f_ Ga_colloid_ = 0, *R*_f_^68^Ga_free_ = 1; mobile phase 2: *R*_f_ [Ga(THP^H^)] = 1, *R*_f_^68^Ga_free_ = 0, ^68^Ga_colloid_ = 0). Radiolabelling of THP^H^ at 0.1 μM was performed using a second E&Z generator, eluted with clinical grade 0.1 M HCl (E&Z).

The ^nat^Ga complexes of THP^H^ and THPO were prepared by addition of an aqueous solution of Ga(NO_3_)_3_ (5 μL, 2 mg mL^–1^, excess) to a solution of the ligand (50 μL, 150 μM) in ammonium acetate 0.2 M. After 5 min reaction time, an aliquot of the reaction mixture was applied to a reversed-phase HPLC column to confirm complex formation (for THP^H^: method 3, for THPO: method 4, see Fig. 4A and B). **[Ga(THP**^**H**^**)] ESI-MS (*m*/*z*):** 764.22 [M + H]^+^, 382.61 [M + 2H]^2+^, 349.66 [M – Ga + 4H]^2+^. Calcd: 764.22 for C_33_H_40_N_7_O_10_Ga + H^+^. The spectrum is reported in Fig. S4.† No peaks assignable to stoichiometry other than 1 : 1 were visible. Solubility of the complex in water or any other solvent was not sufficient to obtain a satisfactory NMR spectrum or X-ray-quality crystals.

### Lipophilicity determination

^68^Ga-radiolabelling of THP^Me^, THP^H^ and THPO (200 μM in ammonium acetate 0.5 M) was performed as described above and verified by iTLC-SG. An aliquot (10 μL) of each radiolabelling mixture was then added to vials containing a pre-equilibrated mixture of octanol/water (500/490 μL) for log *P* measurements, or octanol/PBS (500 μL/490 μL) for log *D*_7.4_ measurements. The mixtures were vortexed and then shaken for 30 min before separation of the two phases by centrifugation (4000 rpm, 3 min). The activity in aliquots of each phase (20 μL aqueous phase, 100 μL octanol phase) was measured in the gamma-counter and corrected for the different volumes sampled. Each experiment was repeated 4 times.

### Spectrophotometric determination of conditional formation constants

The automated titration system consists of a Metrohm 765 Dosimat autoburette, a Mettler Toledo MP230 pH meter with SENTEK pH electrode (P11), and an HP 8453 UV-visible spectrophotometer with a Hellem quartz flow cuvette, with circulation driven by a Gilson Mini-plus #3 pump (speed capability 20 mL min^–1^). A potassium chloride electrolyte solution (0.1 M) was used to maintain the ionic strength. The temperature of the test solutions was maintained in a thermostatic jacketed titration vessel at 25 ± 0.1 °C, using a Fisherbrand Isotemp water bath. The pH electrodes were calibrated using the software GLEE^47^ with data obtained by titrating a volumetric standard HCl (0.1 M) in KCl (0.1 M) with KOH (0.1 M) under an atmosphere of argon. Analytical grade reagent materials were used in the preparation of all solutions. The solution under investigation was stirred vigorously during the experiment. For p*K*_a_ determinations, a cuvette path length of 10 mm was used, while for metal stability constants determinations, a cuvette path length of 50 mm was used (experimental concentration was *ca.* 40 μM for iron complexes and *ca.* 10 μM for gallium complexes). All instruments were interfaced to a computer and controlled by an in-house program.

The automated titration adopted the following strategy: the pH of a solution was increased in increments of 0.1 pH unit by the addition of potassium hydroxide solution (0.1 M) from the autoburette. The pH readings were judged stable if they varied by less than 0.01 pH unit after a pre-set incubation period. For p*K*_a_ determinations, an incubation period of 1.5 min was adopted; for metal stability constant determinations, an incubation period of 3 min was adopted. The cycle was repeated until the predefined end point pH value was achieved. Titration data were analysed with the HypSpec2014 program^48^,^49^ (; http://www.hyperquad.co.uk/). The fitting spectra range for iron complexes was 400–700 nm while that for gallium complexes was 250–350 nm. pH values higher than 11.3 (outside the pH range in which electrode measurements are considered accurate) were neglected and re-calculated from the added KOH quantity (using “no pH” mode within the HypSpec2014 program). The associated hydrolysis constants used in the analysis were collected from Martell's critical stability constants.^50^ Metal affinities of compounds in this study were determined in competition with the metal hydrolysis species in a solution at a high pH (titrated up to pH 12.5). Satisfactory fitting of the THP^Me^ titration for both iron and gallium were achieved. In contrast, the titration of THP^H^ was found to be more complex due to the presence of the additional protonation sites. A satisfactory result for the THP^H^/gallium interaction was achieved, but not for iron, which is possibly related to different log stability constants of [Fe(OH)_4_]^–^ and [Ga(OH)_4_]^–^ (log *β* = 34.4 and 39.4 respectively^50^). Instead, the corresponding stability constant was obtained using EDTA-iron-hydroxide species competition at the high pH range following a slightly modified titration procedure (cuvette path length: 100 mm, incubation period: 30 min, experimental concentration of iron complexes: *ca.* 20 μM, experimental concentration of EDTA: *ca.* 50 mM). Speciation plots (Tables S1–S3†) were calculated with the HYSS program.^51^

### Ligand competition for ^68^Ga^3+^ binding

A solution containing both THP^Me^ and THP^H^, each 2 mM, in D_2_O was diluted to 100 μM with aqueous ammonium acetate (0.5 M) after NMR analysis had been used to confirm equal concentration of the two compounds (Fig. S5†). 50 μL of the mixture were mixed with an equal volume of ^68^Ga eluate. The percentage of radioactivity associated with each compound was measured by iTLC-SG (mobile phase 1 and 2) at different time points (1, 5, 10, 30, 60 and 120 minutes).

### Metal competition for THP binding

A standard ICP-MS solution of iron(iii) in nitric acid (Alfa Aesar) was diluted in 0.1 M nitric acid (Fluka analytical) to obtain 2250 μM and 225 μM Fe(iii) solutions. The same procedure was used to prepare a 225 μM Ga(iii) solution from its ICP-MS standard solution (Sigma Aldrich). The Ga(iii) solution (20 μL, 225 μM) was mixed with the Fe(iii) solution (20 μL, 225 or 2250 μM Fe) or with 0.1 M nitric acid as a control (20 μL) and the ^68^Ga generator eluate was added (20 μL, [Ga^3+^] ≈2 nM and was considered negligible). The relevant THP ligand (440 μL of a 11.3 μM solution in 0.5 M ammonium acetate buffer) was added to the radiolabelling mixture (final concentration: [THP] = 10 μM, [Ga^3+^] = 9 μM, [Fe^3+^] = 0, 9 or 90 μM). The radiochemical yield at different times was measured by iTLC-SG (mobile phase 2). Statistical analysis was performed using a *t*-test between the “no Fe” and “0.9 eq. Fe” groups.

### [^67^Ga(THP^H^)] serum stability

^67^Ga chloride (50 μL, 5 MBq), obtained from Nordion (Canada), was added to THP^H^ (50 μL, 100 μM) in 0.5 M ammonium acetate. Quantitative radiolabelling was verified after 5 min of incubation, *via* iTLC-SG. The retention time on a size-exclusion HPLC (mobile phase: PBS with 50 mM EDTA trisodium salt) was 15 min 30 s for [^67^Ga(THP^H^)]. The retention time for ^67^Ga chloride in the same conditions was 12 min. An aliquot of each solution (60 μL) was added to male AB human serum (600 μL, Sigma) and incubated at 37 °C. Size-exclusion radiochromatography was performed after 1 h, 1 day and 8 days incubation.

### *In vivo* studies with THP^H^

All *in vivo* experiments were carried out in accordance with British Home Office regulations governing animal experimentation and complied with guidelines on responsibility in the use of animals in bioscience research of the U.K. Research Councils and Medical Research Charities, under U.K. Home Office project and personal licences. Male SCID/beige mice (7 months old, Charles River) were used for preliminary animal studies on THP^H^. Dynamic PET scanning was performed using a nanoScan® PET/CT (Mediso Medical Imaging Systems).^52^ Respiration rate and bed temperature were monitored throughout. PET/CT datasets were reconstructed using the Monte-Carlo-based full 3D iterative algorithm Tera-Tomo (Mediso Medical Imaging Systems).^53^ All reconstructed datasets were analysed using VivoQuant 1.21 software (inviCRO), which enables the co-registration of PET and CT images and the delineation of regions of interest (ROIs) for quantification of activity in specific organs. Mice were anaesthetised with isoflurane (O_2_ flow rate of 1.0–1.5 L min^–1^ and isoflurane levels of 2–2.5%) cannulated at the tail vein using a catheter (25 μL volume) and a CT scan was performed. Subsequently, a PET scan was started and the radiotracer injected. One mouse (37 g) was injected with of a [^68^Ga(THP^H^)] solution (307 μL, 5 μM, 6.59 MBq) and imaged for 1 hour to determine biodistribution and *in vivo* stability of the radiolabelled complex. A second mouse (33 g) was injected with acetate buffered ^68^Ga (100 μL, 0.1 M ammonium acetate, 4.68 MBq), without THP^H^ or other chelator, while scanning, followed at 30 min by an injection of THP^H^ (50 μL of a 50 μM solution in PBS). Animals were then sacrificed by neck dislocation while still anaesthetised. Urine was collected and analysed by reversed-phase HPLC (method 3).

## Authors contribution

All authors have given approval to the final version of the manuscript.

## Abbreviations

CT: Computed tomography
DCC: Dicyclohexylcarbodiimide
EDTA: Ethylenediaminetetraacetic acid
FSC: Fusarinine-C
HBED: *N*,*N*-Bis(2-hydroxybenzyl)ethylenediamine-*N*,*N*-diacetic acid
HOBT: Hydroxybenzotriazole
HP: 3-Hydroxypyridin-4-one
HPLC: High performance liquid chromatography
iTLC-SG: Instant thin-layer chromatography-silica gel
MIP: Maximum intensity projection
PBS: Phosphate-buffered saline
PET: Positron emission tomography
PSMA: Prostate specific membrane antigen
RCY: Radiochemical yield
SSTR2: Somatostatin receptor type II
THP: Tris(3-hydroxypyridin-4-one)
THPO: Tris(6-methyl-3-hydroxypyran-4-one)

## Conflicts of interest

P. J. B. and R. C. H. are named inventors on related patents. All other authors have no conflicts to declare.

## Supplementary Material

Supplementary information
