# Supplementary material for: Tuning the properties of tris(hydroxypyridinone) ligands: efficient 68Ga chelators for PET imaging
Source: Dalton Trans. 2019 Mar 12;48(13):4299–313. doi: 10.1039/c8dt04454f (PMC6469224; doi:10.1039/c8dt04454f)
Supplement: Supplementary file 1 [file DT-048-C8DT04454F-s001.pdf]

Electronic Supplementary Material (ESI) for Dalton Transactions.
This journal is © The Royal Society of Chemistry 2019

Electronic Supplementary Information

Tuning the properties of *tris*(hydroxypyridinone) ligands: efficient ^{68}Ga chelators for PET imaging

Content

S.1 THP^{Me} radiolabelling

S.2 THP^{Me} *in vivo* radiolabelling experiment

Table S1-S2 Measured complex formation constants for THP^{Me} and THP^{H}

Figure S1 Chemical structures of selected Ga^{3+} chelators evaluated for use with ^{68}Ga.

Figure S2, S3 HPLC characterisation of THP^{H} and THPO (additional UV chromatograms)

Figure S4 [^{nat}Ga(THP^{H})] mass spectrum

Figure S5 Absorbance spectra of THP ligands

Figure S6 ^{1}H NMR spectrum of an equimolar mixture of THP^{H} and THP^{Me}

Figure S7 Serum stability of [^{67}Ga(THP^{H})]

Figure S8 *In vivo* behaviour of THP^{Me}

Figure S9-S16 ^{1}H NMR and ^{13}C NMR of compounds **2-9**

S.1 THP^{Me} radiolabelling

An E&Z generator was eluted with 5 mL of pure HCl 0.1 M (Fluka analytical) in five 1 mL fractions and their activity measured by a Capintec radionuclide dose calibrator. An aliquot of the highest activity fraction (100 µL, 15-20 MBq) was mixed with THP^{Me} (100 µL, concentration range 200-2 µM) in ammonium acetate 0.5 M. Verification of the radiolabelling was carried out after 5 minutes by reversed-phase HPLC (method 7: Mobile phase A: H_2O + 0.1% TFA, B: MeCN + 0.1% TFA, % B increased from 2% at 5 minutes to 90% at 25 minutes and back to 2% at 27 minutes) and iTLC-SG (mobile phase 1: R_f [Ga(THP^{Me})] = 0, R_f $Ga_{colloid}$ = 0, R_f $^{68}Ga_{free}$= 1; mobile phase 2: R_f [Ga(THP^{Me})] = 1, R_f $^{68}Ga_{free}$ = 0, $^{68}Ga_{colloid}$ = 0). Quantitative radiolabelling was obtained for all examined concentrations. A single peak was obtained in the radiochromatogram at 15 min 28 s (**Figure S8B,** upper panel). Radiolabelling of THP^{Me} at 0.1 µM was performed using a second E&Z generator, eluted with clinical grade 0.1 M HCl (E&Z).

S.2 THP^{Me} *in vivo* radiolabelling experiment

A female BALB/c mouse (22 g, 9 weeks, Charles Rivers) was anaesthetised with isoflurane (O_2 flow rate of 1.0-1.5 L/min and isoflurane levels of 2-2.5%), cannulated using a catheter (25 µL volume) and subjected to a CT scan. Subsequently, a PET scan was started, and the mouse injected with acetate buffered ^{68}Ga (150 µL, 0.1 M ammonium acetate, 8.12 MBq) while scanning, followed at 45 minutes by an injection of THP^{Me} (100 µL of a 24 µM solution in PBS). Upon completion of the scans, the animal was sacrificed by neck dislocation whilst anaesthetised and the urine was collected and analysed by reversed-phase HPLC (method 7). Similar to what was observed for THP^{H}, THP^{Me} was able to bind ^{68}Ga *in vivo* thus accelerating its blood clearance (**Figure S8A**), and HPLC analysis of mouse urine (**Figure S8B**) revealed quantitative formation of the [^{68}Ga(THP^{Me})] complex. These data confirm previous *in vitro* studies demonstrating the ability of THP^{Me} to transchelate transferrin-bound Ga(III).[1]

Table S1. *Formation constants for the Ga(III) and Fe(III) complexes of deferiprone. Detailed species and chemical equations for which the complex formation constants were determined are defined below. Speciation plots for* $[L]_{total}$ *= 10 µM and* $[M]_{total}$ *= 1 µM are also reported.*

Deferiprone

L= 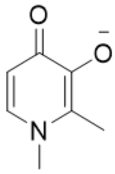

Chemical equilibrium equations

$M^{3+} + L^{-} \rightleftarrows [ML]^{2+}$	$Log\ \beta_{[ML]^{2+}}$
$M^{3+} + 2\,L^{-} \rightleftarrows [ML_2]^{+}$	$Log\ \beta_{[ML_2]^{+}}$
$M^{3+} + 3\,L^{-} \rightleftarrows [ML_3]$	$Log\ \beta_{[ML_3]}$
$M^{3+} + 4\,OH^{-} \rightleftarrows [M(OH)_4]^{-}$	$Log\ \beta_{[M(OH_4)]^{-}}$

Complex formation constants

$Log\ \beta_{[FeL]^{2+}}$	15.0 *	$Log\ \beta_{[GaL]^{2+}}$	14.4 *
$Log\ \beta_{[FeL_2]^{+}}$	27.5 *	$Log\ \beta_{[GaL_2]^{+}}$	27.0*
$Log\ \beta_{[FeL_3]}$	37.5 *	$Log\ \beta_{[GaL_3]}$	37.4 *
$Log\ \beta_{[Fe(OH_4)]^{-}}$	34.4 [2]	$Log\ \beta_{[Ga(OH_4)]^{-}}$	39.4 [2]
pFe	20.8	pGa	20.7

Speciation plots

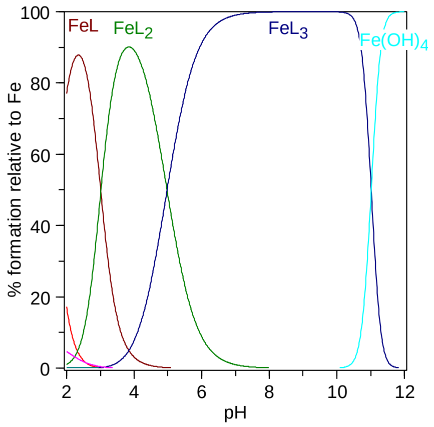

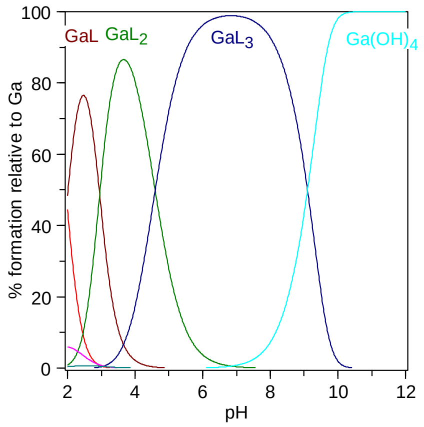

* Stability constants for deferiprone have been previously reported in the literature;[3] measurements have been repeated here as a control to verify the accuracy of our titration methods.

Table S2. *Conditional formation constants for the Ga(III) and Fe(III) complexes of THP^{Me}. Detailed species and chemical equations for which the constants were determined are defined below. Speciation plots for $[L]_{total}$ = 10 µM and $[M]_{total}$ = 1 µM are also reported.*

**THP^{Me}**			
L^{3-} = 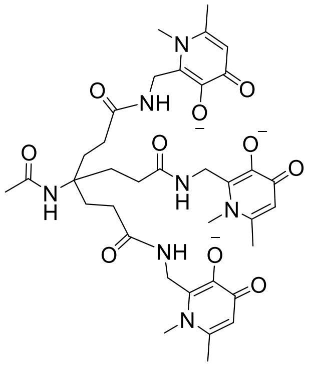			
**Chemical equilibrium equations**			
$M^{3+} + L^{3-} \rightleftarrows [ML]$		$Log\ \beta_{[ML]}$	
$M^{3+} + 4\ OH^{-} \rightleftarrows [M(OH)_4]^{-}$		$Log\ \beta_{[M(OH_4)]^{-}}$	
**Conditional formation constants**			
$Log\ \beta_{[FeL]}$	34.2	$Log\ \beta_{[GaL]}$	35.0
$Log\ \beta_{[Fe(OH_4)]^{-}}$	34.4[2]	$Log\ \beta_{[Ga(OH_4)]^{-}}$	39.4[2]
pFe	29.1	pGa	30.0
**Speciation plots**			
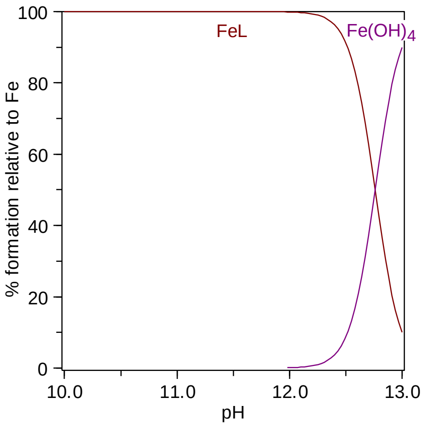		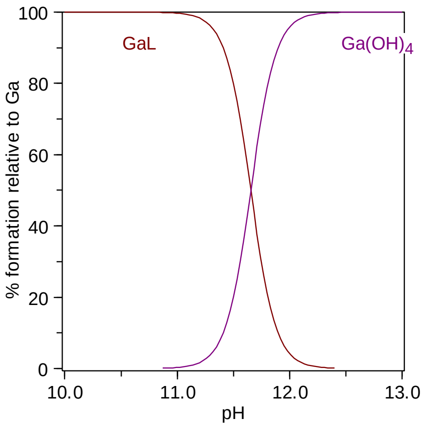	

Table S3. *Conditional formation constants for the Ga(III) and Fe(III) complexes of THP^H. Detailed species and chemical equations for which the constants were determined are defined below. Speciation plots are also reported for $[L]_{total}$ = 10 μM and $[M]_{total}$ = 1 μM (gallium complex) and $[L]_{total}$ = 20 μM, $[M]_{total}$ = 20 μM and $[EDTA]_{total}$ = 50 mM (iron complex).*

THP^H

$[LH_3]^{3-}$ =

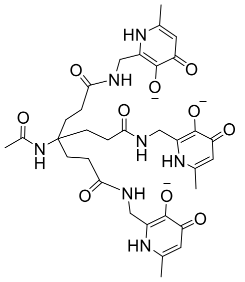

L^{6-} =

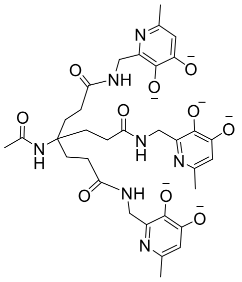

Chemical equilibrium equations

$M^{3+} + L^{6-} + 3\,H^+ \rightleftarrows [MLH_3]$	$Log\ \beta_{[MLH_3]}$*
$M^{3+} + L^{6-} \rightleftarrows [ML]^{3-}$	$Log\ \beta_{[ML]^{3-}}$
$M^{3+} + 4\,OH^- \rightleftarrows [M(OH)_4]^-$	$Log\ \beta_{[M(OH_4)]^-}$
$Fe^{3+} + EDTA^{4-} + 2\,OH^- \rightleftarrows [FeEDTA(OH)_2]^{3-}$	$Log\ \beta_{[FeEDTA(OH)_2]^{3-}}$

Conditional formation constants

$Log\ \beta_{[FeLH_3]}$	72.9*	$Log\ \beta_{[GaLH_3]}$	76.2*
$Log\ \beta_{[FeL]^{3-}}$	40.9	$Log\ \beta_{[GaL]^{3-}}$	42.5
$Log\ \beta_{[Fe(OH_4)]^-}$	34.4[2]	$Log\ \beta_{[Ga(OH_4)]^-}$	39.4[2]
$Log\ \beta_{[FeEDTA(OH)_2]^{3-}}$	42.0[2]	pGa	31.8
pFe	28.6		

Speciation plots

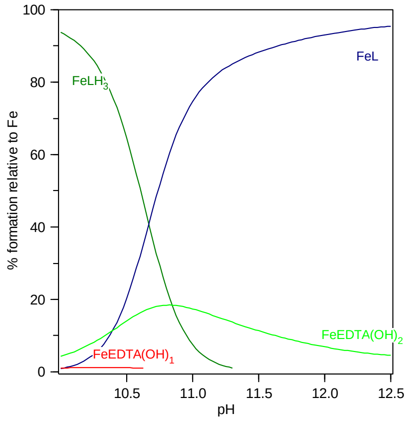

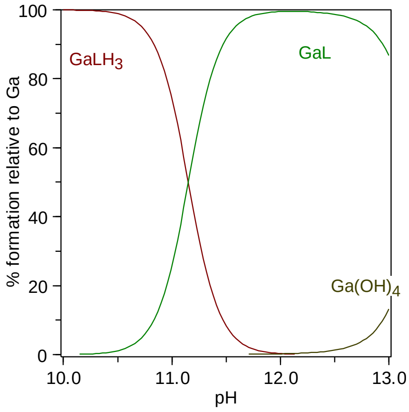

* Because only intrinsic metal complex protonation[4] can be discriminated using this method, the cumulative 3 protons metal complex formation constant was fitted with the data with no further stepwise constant fitting.

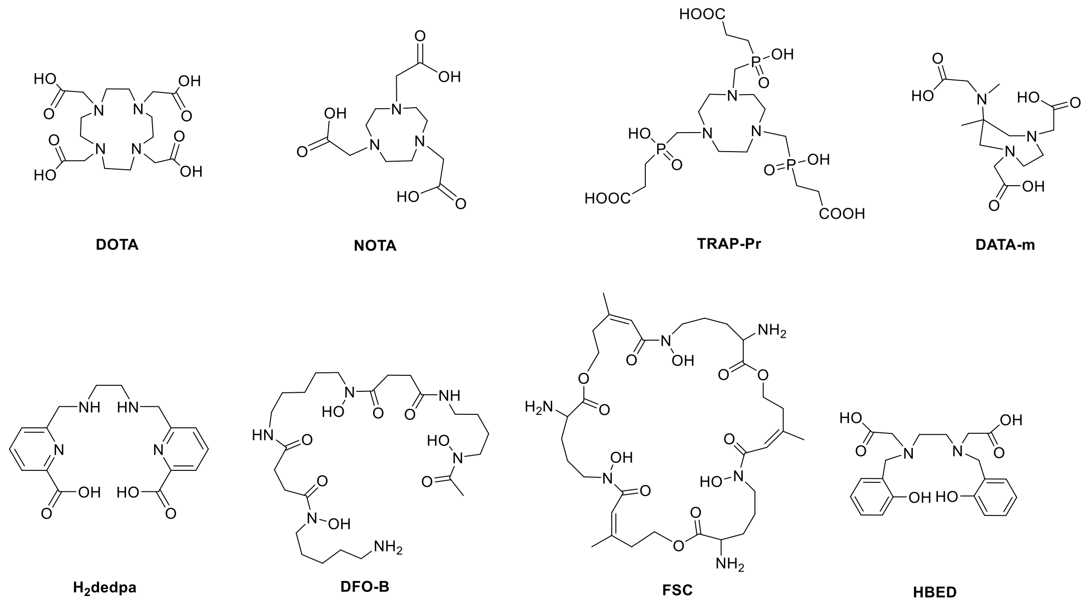

Figure S1. *Chemical structures of selected Ga^{3+} chelators evaluated for use with ^{68}Ga.*

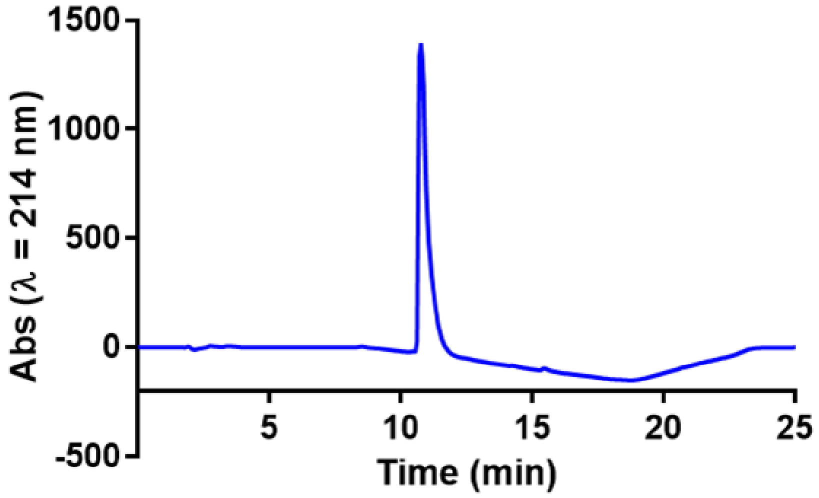

Figure S2. *UV Chromatogram (λ = 214 nm) of THP^{H} using HPLC method 5.*

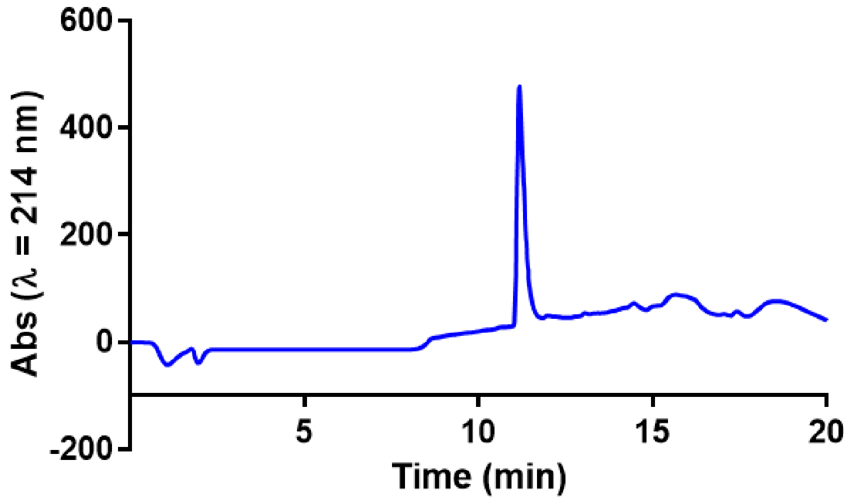

Figure S3*. UV Chromatogram (λ = 214 nm) of THPO using HPLC method 6.*

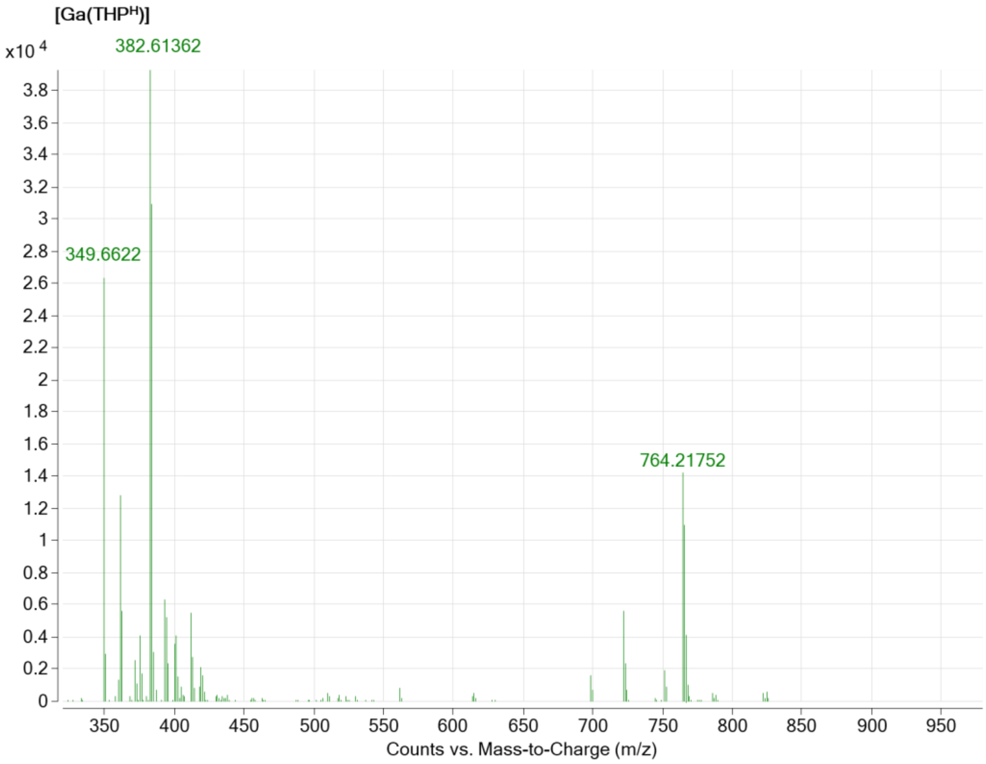

Figure S4. *ESI mass spectrum for the [Ga(THP^{H})] complex. The peak at 764.22 m/z represents [Ga(THP^{H})+H]$^{+}$ and shows the typical isotopic pattern of a natural gallium complex. Peak at 382.61 and 349.67 are assigned to the doubly-charged species [Ga(THP^{H})+2H]$^{2+}$ and [THP^{H}+2H]$^{2+}$, respectively. The minor peak at 698.32 represents [THP^{H}+H]$^{+}$, peaks at 720.29 and 751.23 m/z are assigned to the sodium and iron complex of THP^{H}, respectively. No peaks assignable to stoichiometry other than 1:1 were visible.*

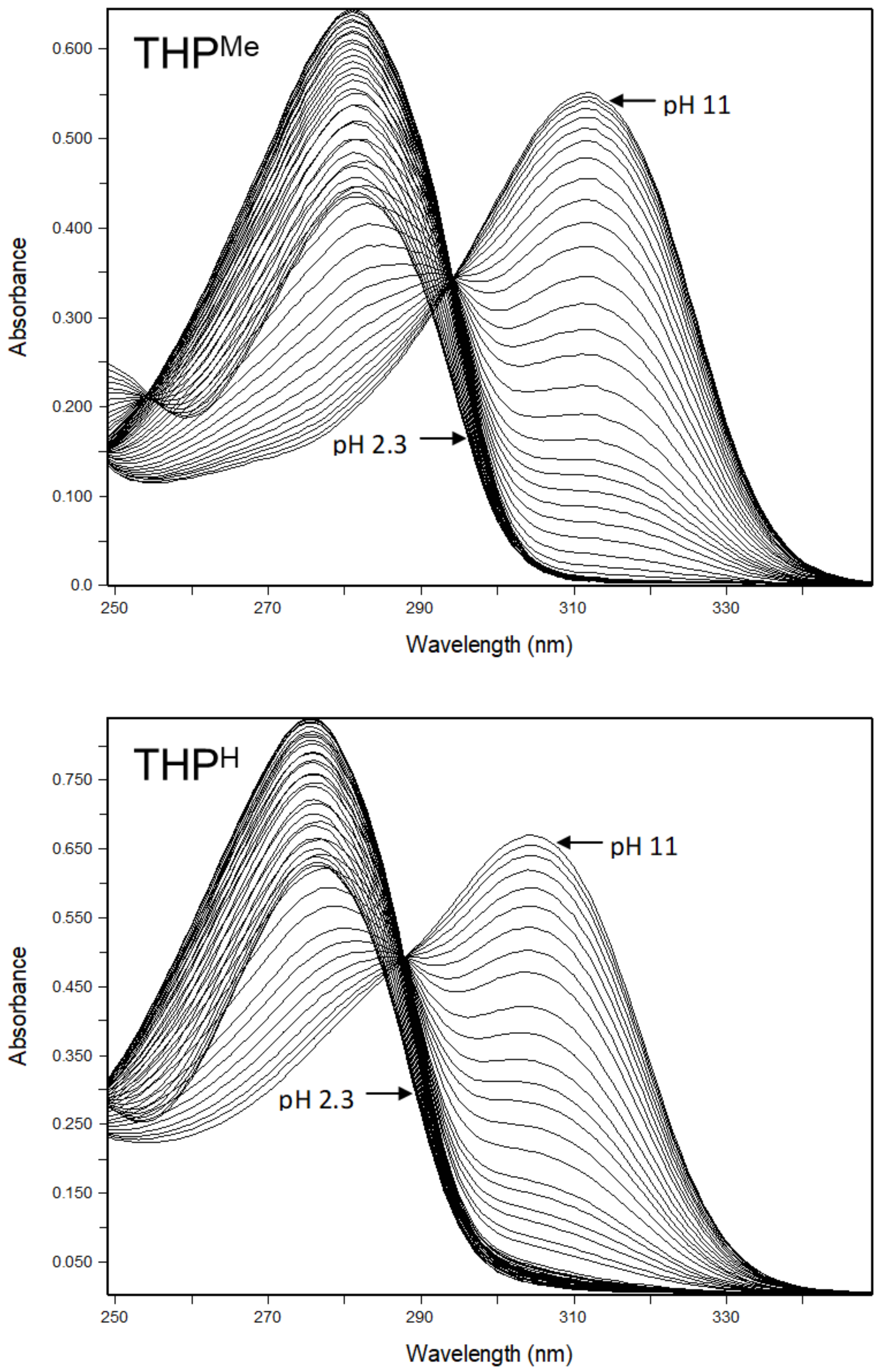

Figure S5. *Absorbance spectra of THP^{Me} and THP^{H} (λ_{range} = 250-400 nm) at different pH values (intervals of 0.1 pH unit).*

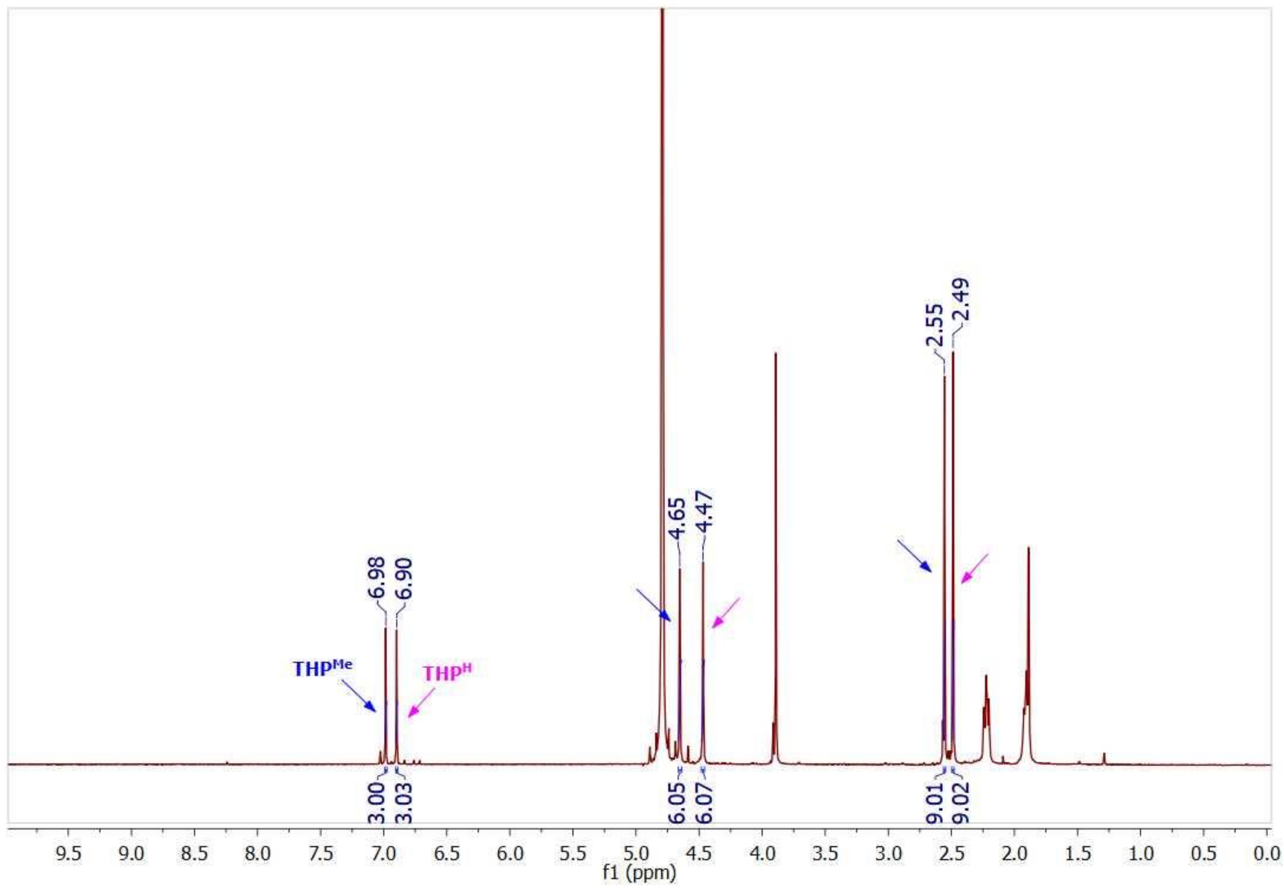

Figure S6. *^{1}H NMR of a solution 2 mM in THP^{Me} and 2 mM in THP^{H} in D_2O. Peaks are referenced to residual solvent signal. Pink and blue arrows indicate signals from THP^{H} and THP^{Me}, respectively. Peak integration is reported (from left to right) for the pyridinone **C^5-H** hydrogen, the C^2-**CH_2**-NH_2 group and the C^6-**CH_3**, confirming equal concentration of the two ligands.*

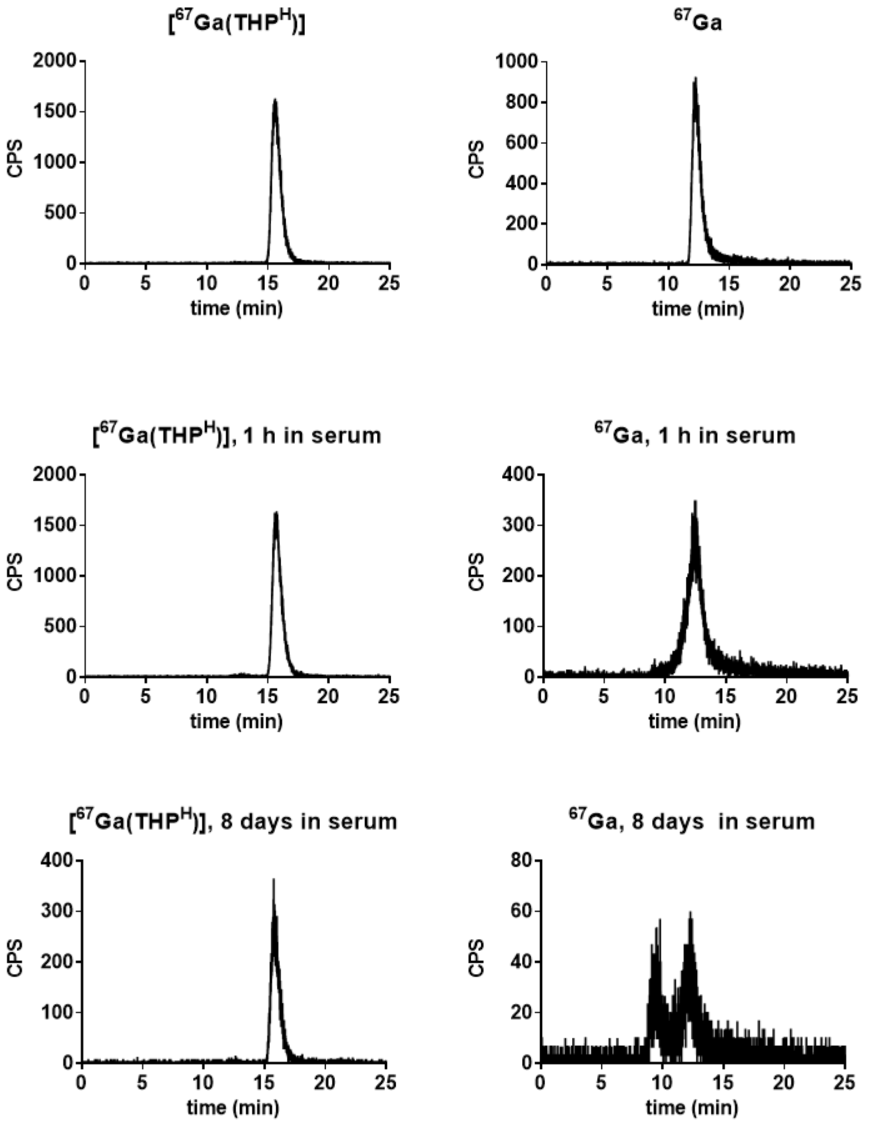

Figure S7. *Size-exclusion radiochromatograms of [^{67}Ga(THP^{H})] and ^{67}Ga without human serum and after incubation with human serum at 37 °C at 1 hour and up to 8 days.*

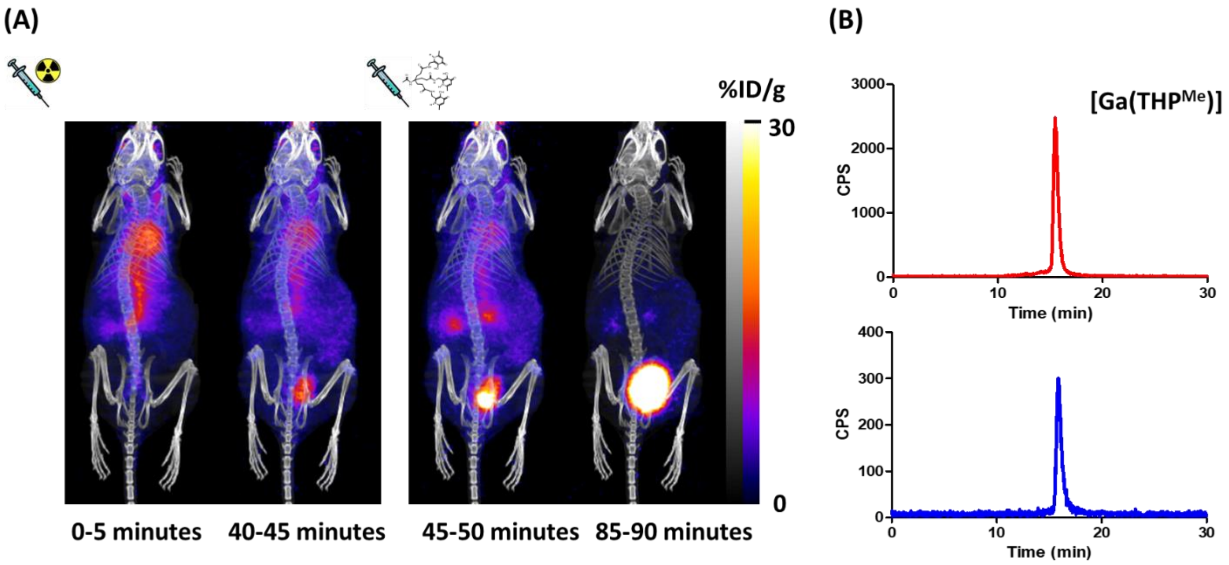

Figure S8. *A) Dynamic PET/CT MIP, at different time points, of a mouse injected with acetate buffered ^{68}Ga at time 0 followed by THP^{Me} at 45 minutes. A sudden change in the biodistribution of ^{68}Ga is visible soon after injection of the chelator, with the blood pool activity quickly clearing through the kidneys into the bladder.* ***B)*** *Comparison of the radiochromatograms of [^{68}Ga(THP^{Me})] (top panel) and of the mouse urine at 90 minutes after ^{68}Ga injection (bottom panel), demonstrating in vivo formation of [^{68}Ga(THP^{Me})] following THP^{Me} administration (reversed-phase HPLC, method 3).*

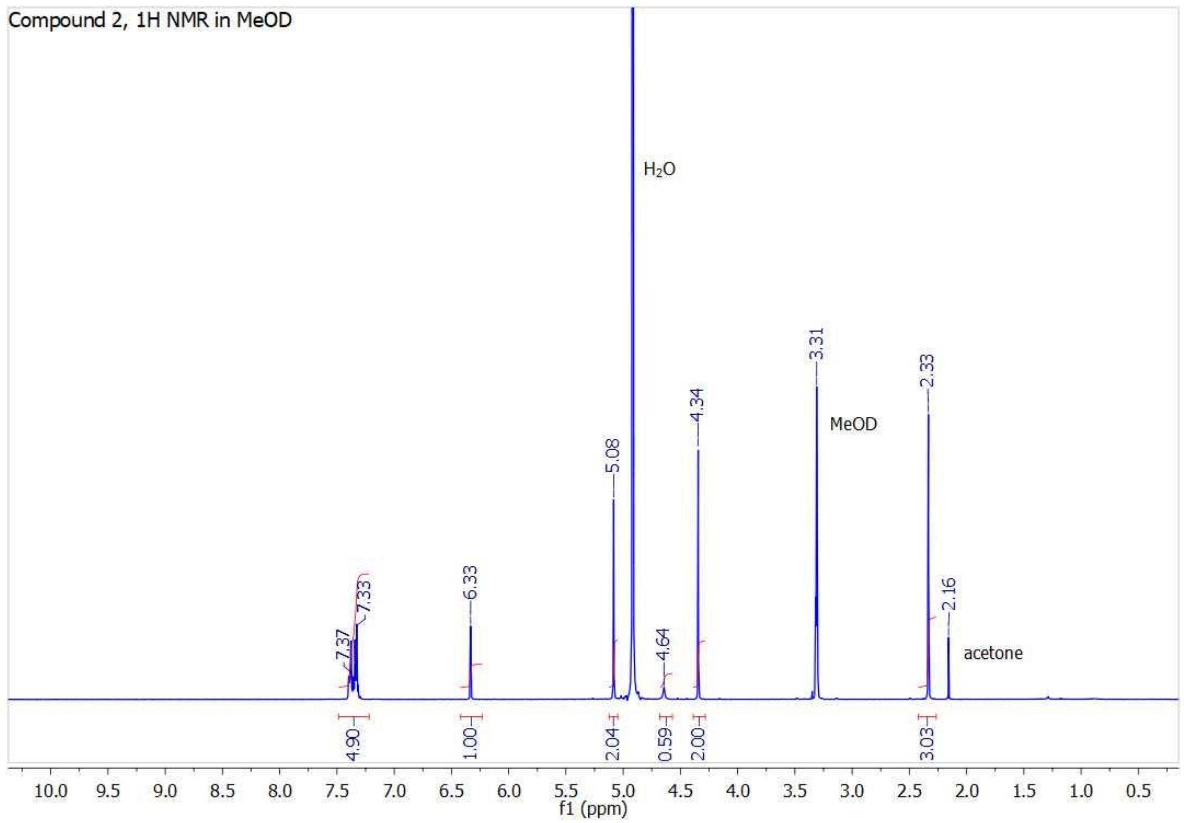

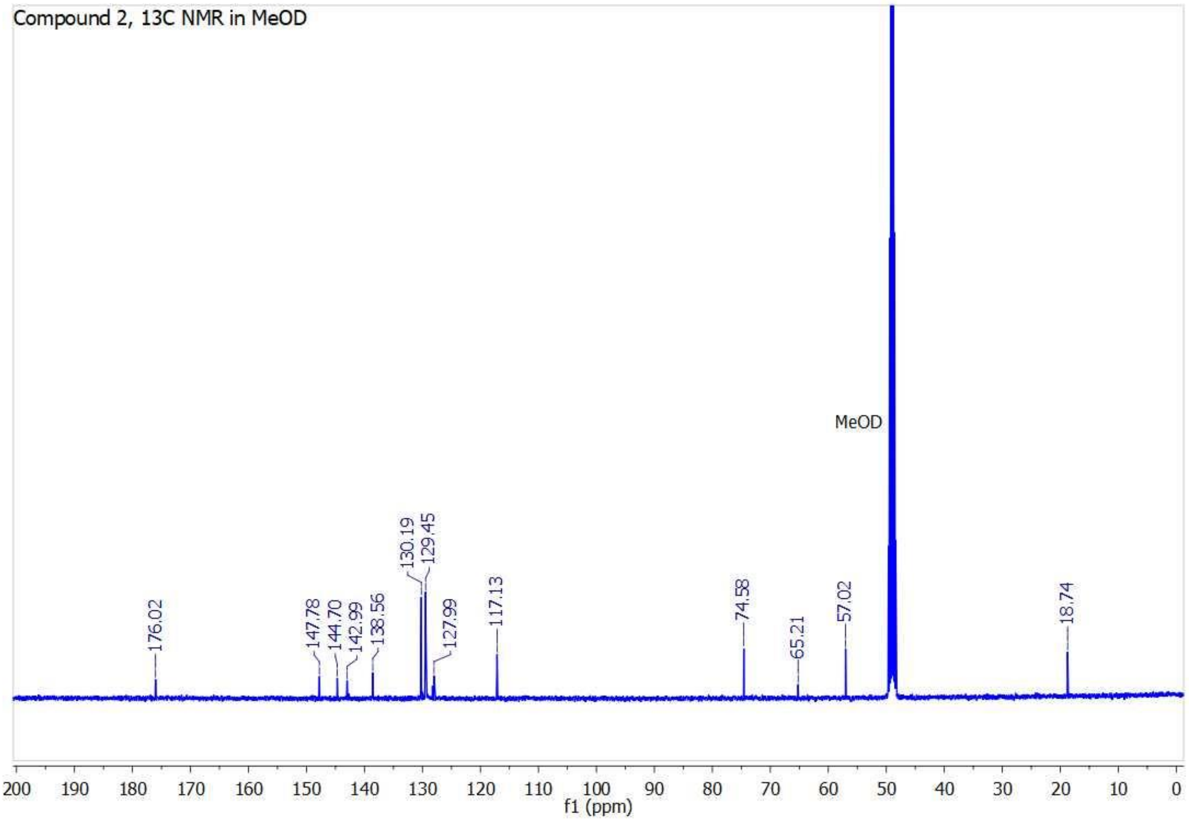

Figure S9. *^{1}H NMR (top panel) and ^{13}C NMR (bottom panel) of* ***2*** *in methanol-d4.*

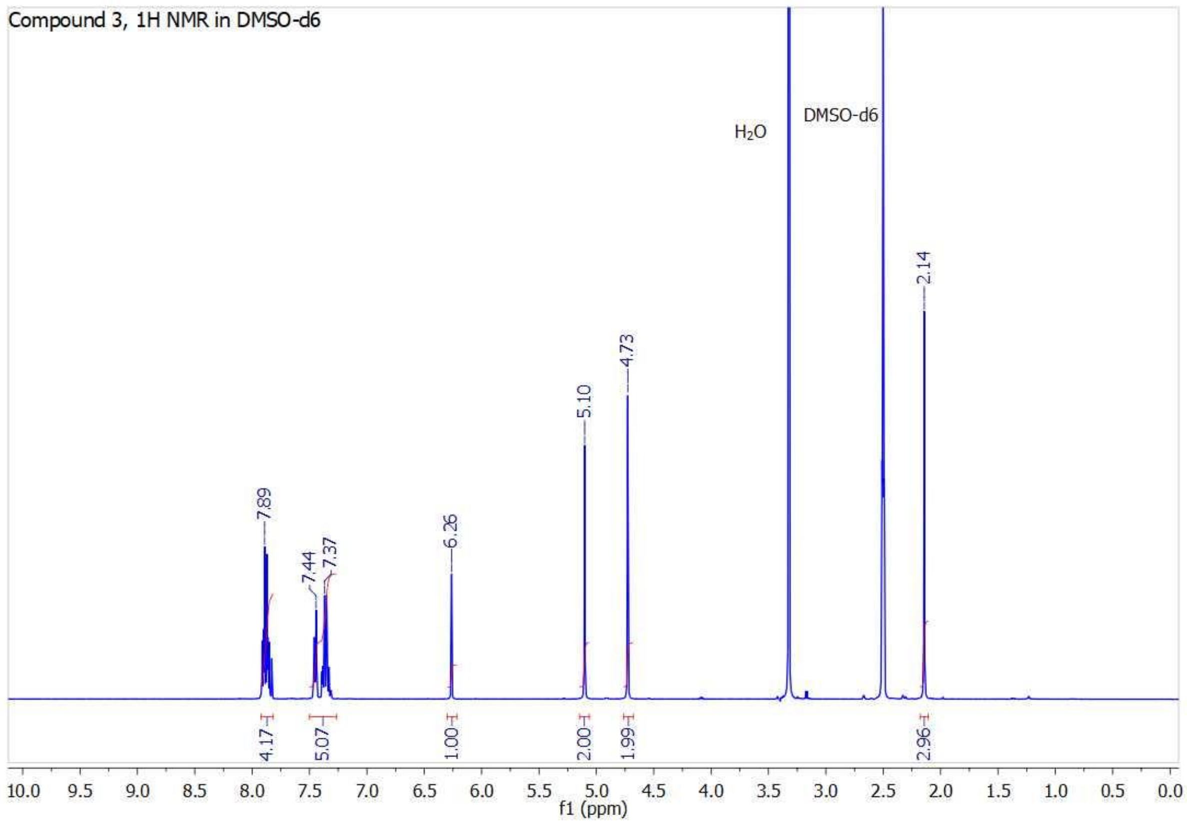

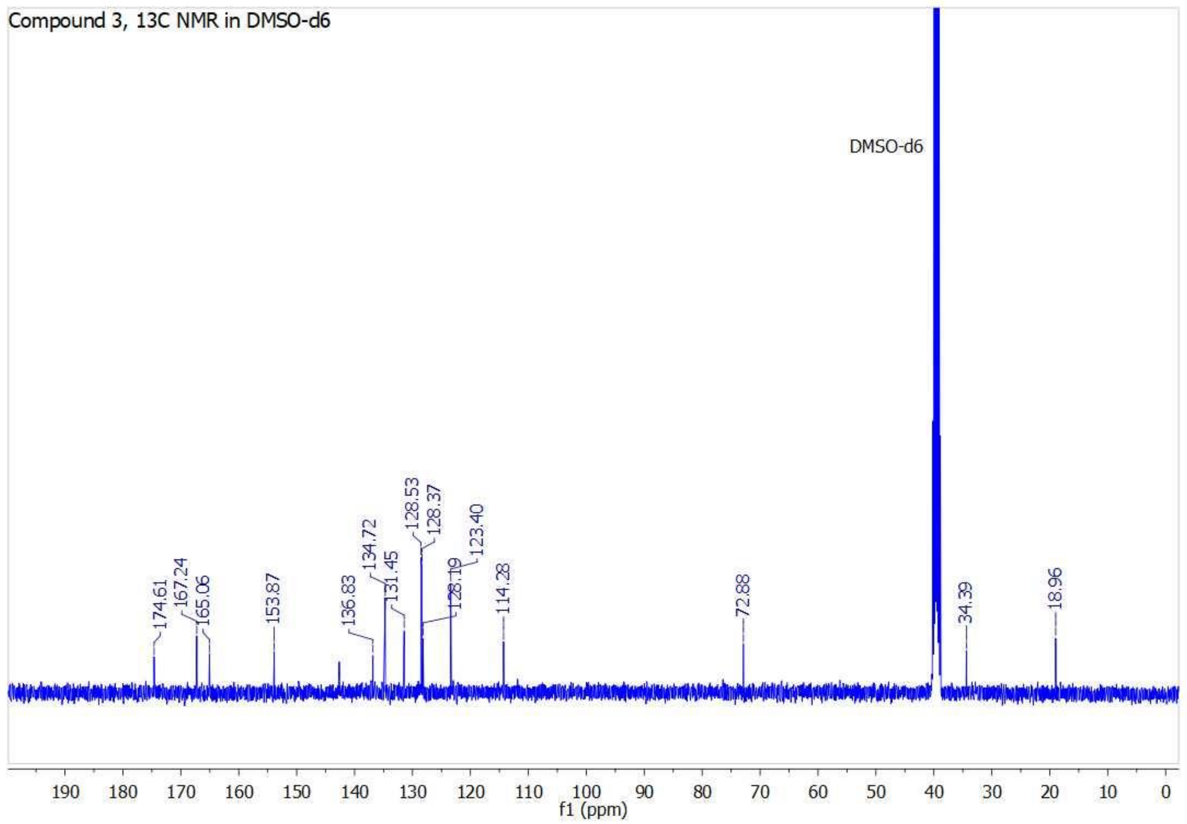

Figure S10. *^{1}H NMR (top panel) and ^{13}C NMR (bottom panel) of **3** in DMSO-d6.*

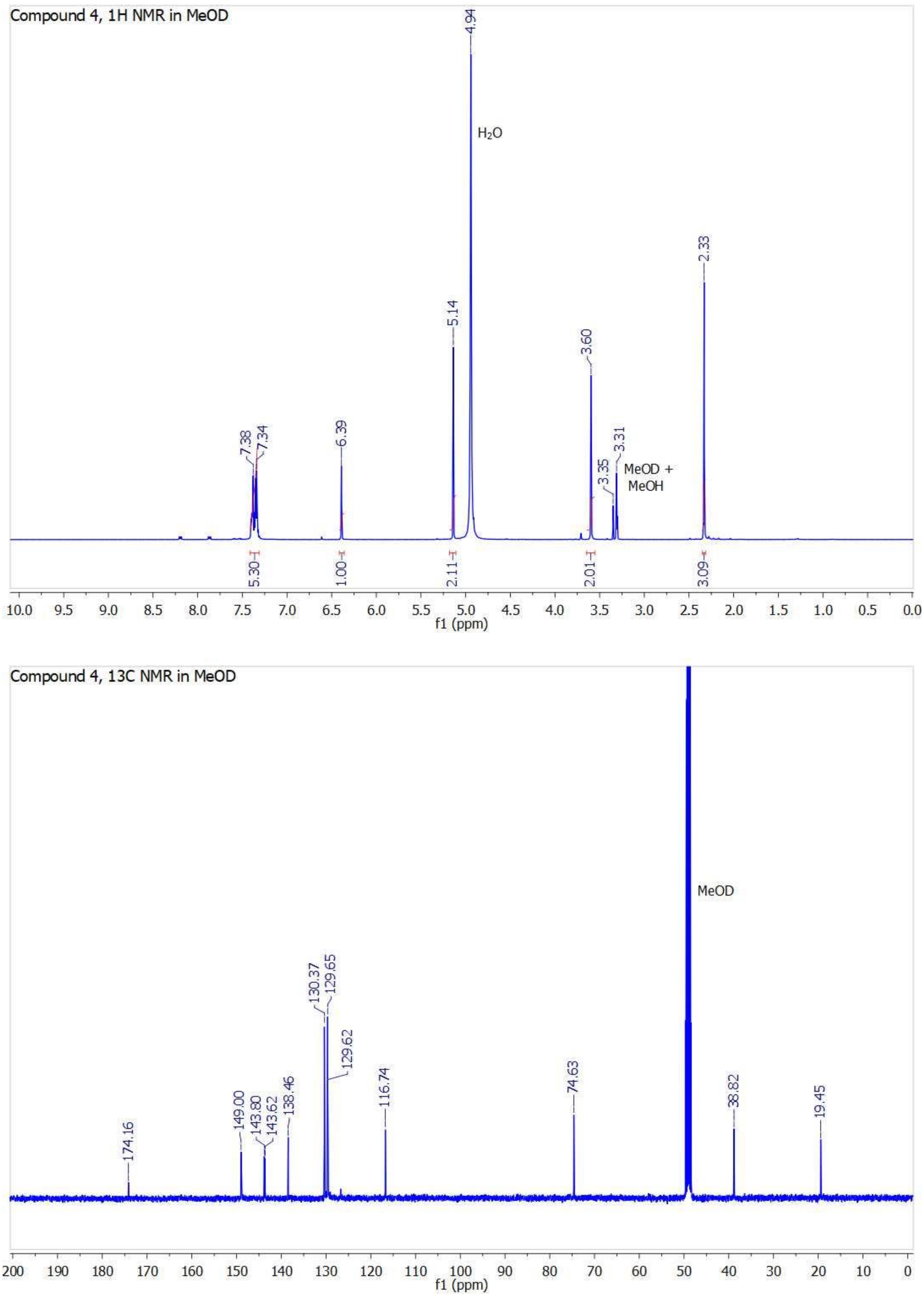

Figure S11. ^{1}H *NMR (top panel) and* ^{13}C *NMR (bottom panel) of* ***4*** *in methanol-d4.*

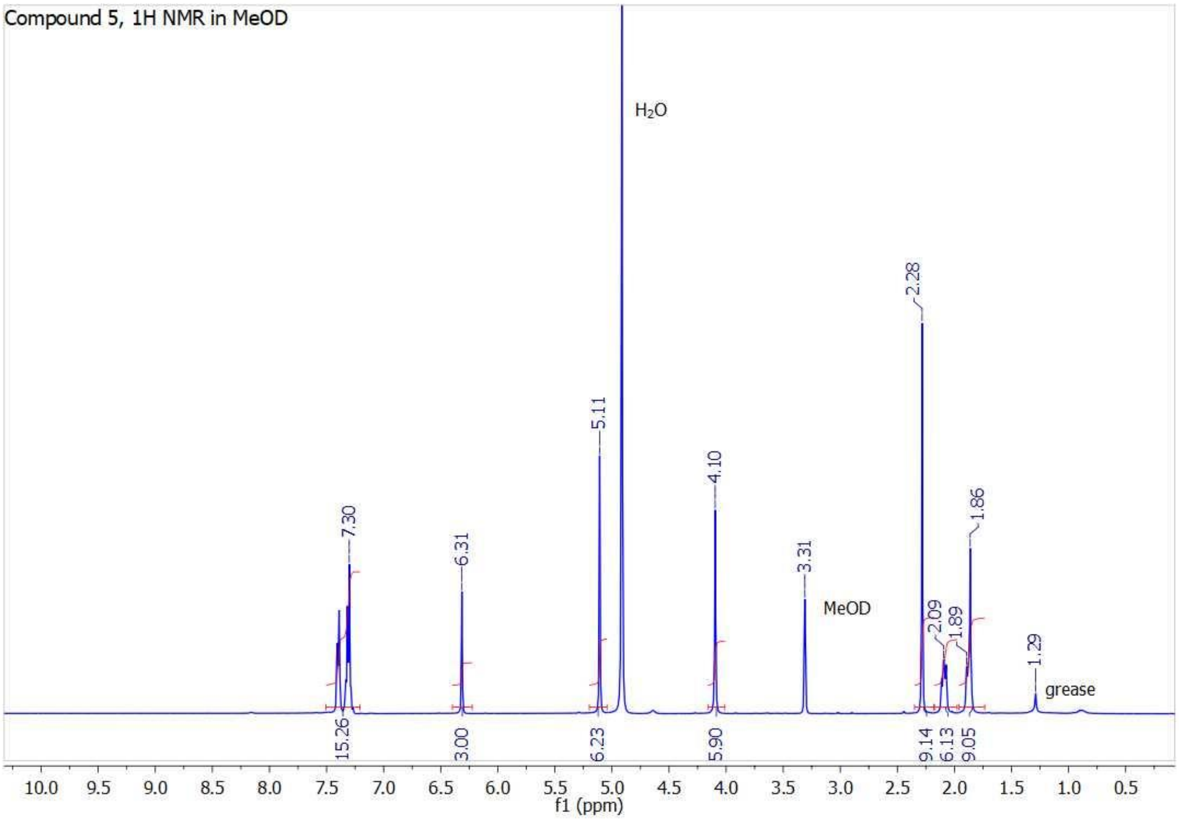

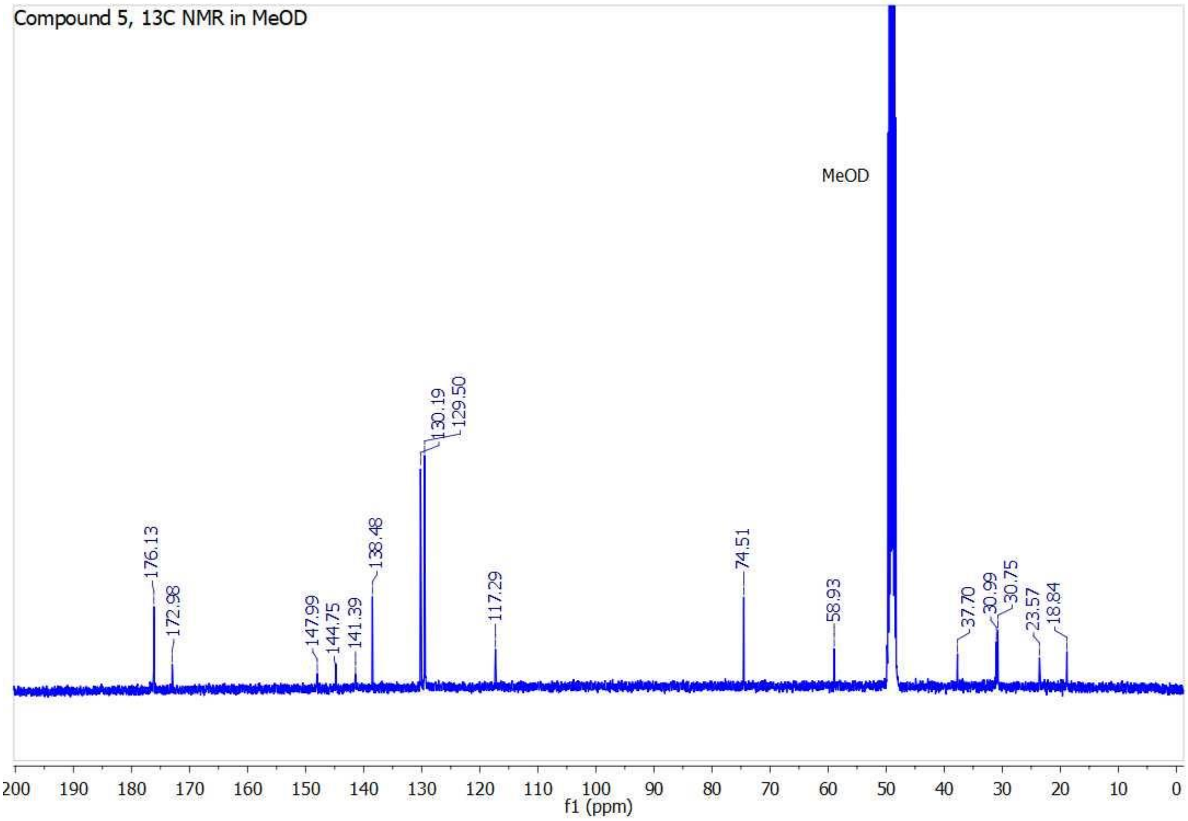

Figure S12. *^{1}H NMR (top panel) and ^{13}C NMR (bottom panel) of **5** in methanol-d4.*

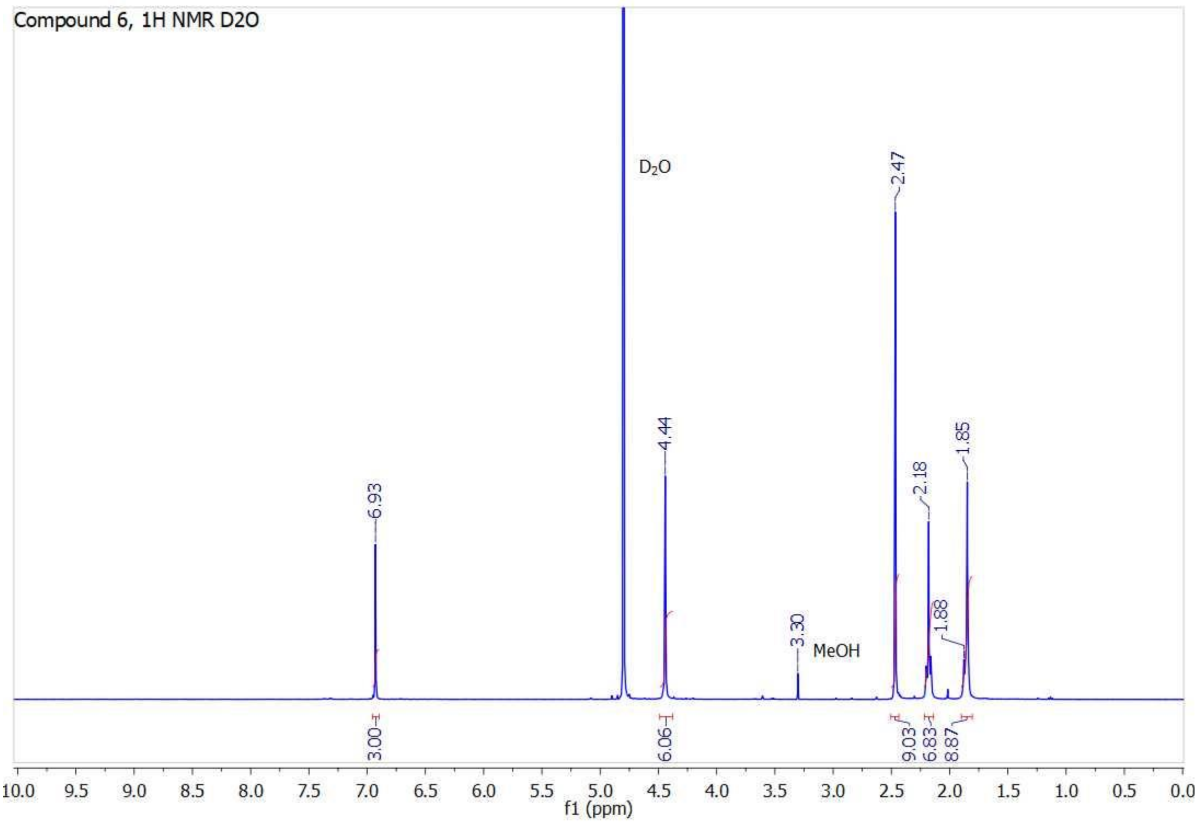

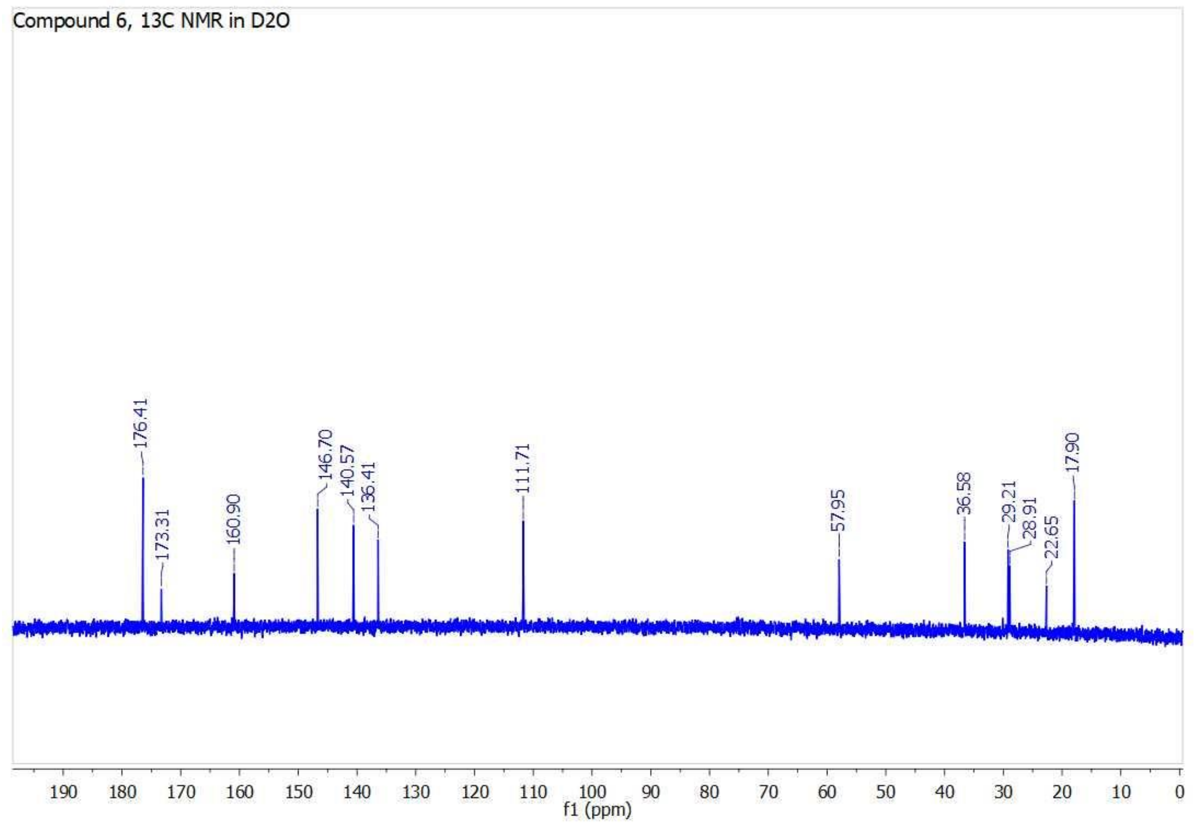

Figure S13. *^{1}H NMR (top panel) and ^{13}C NMR (bottom panel) of* ***6*** *in* D_2O.

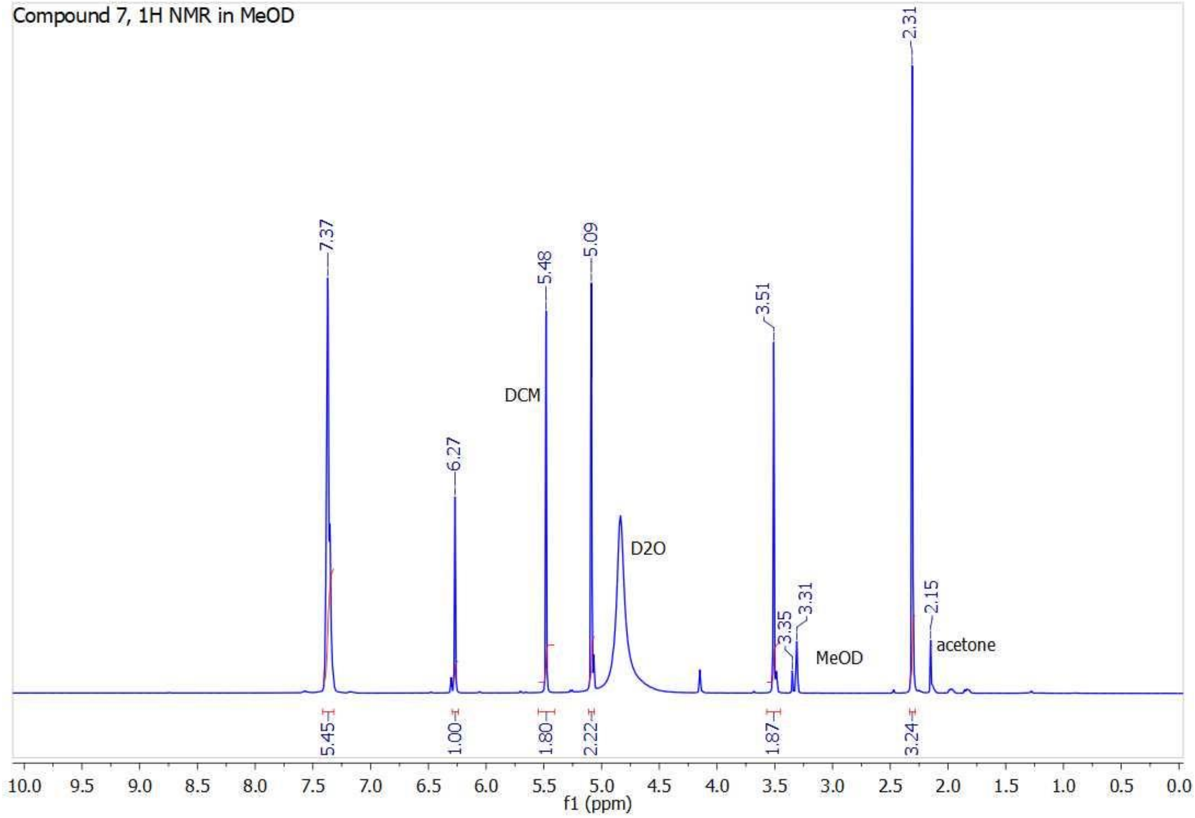

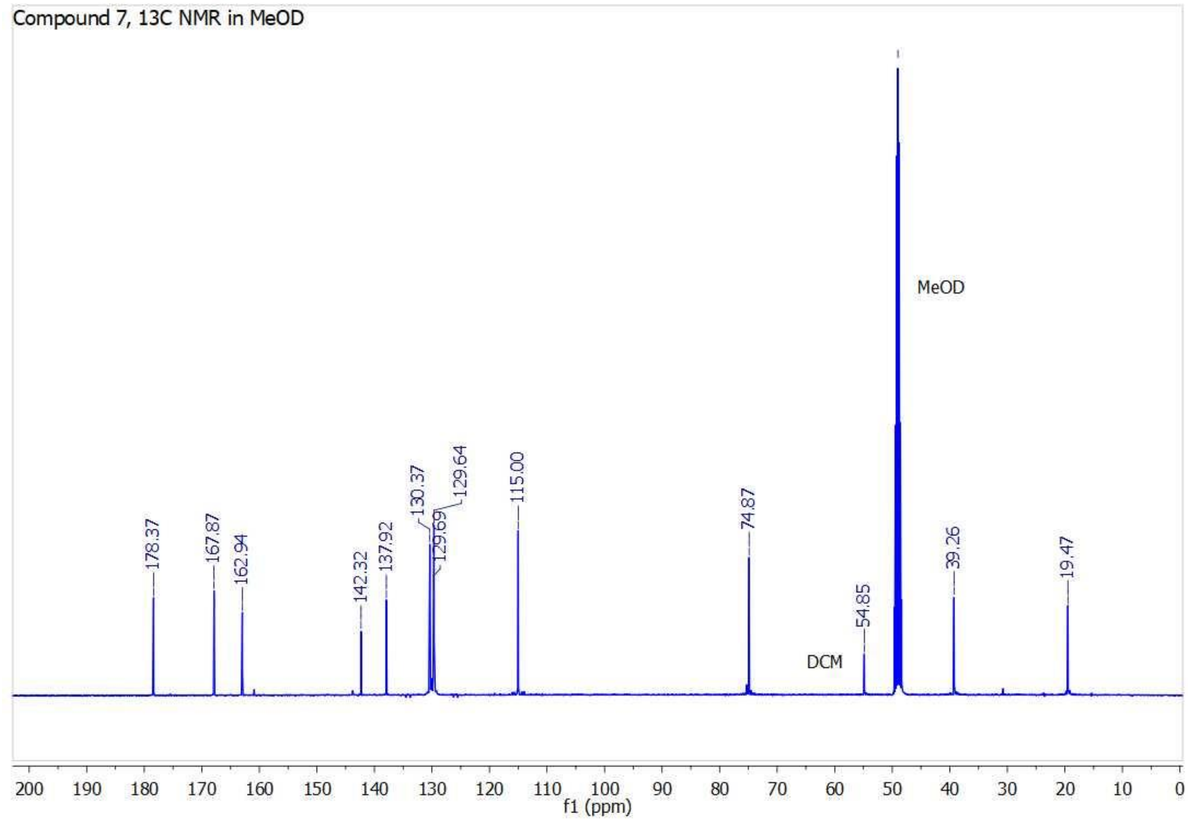

Figure S14. *^{1}H NMR (top panel) and ^{13}C NMR (bottom panel) of **7** in methanol-d4.*

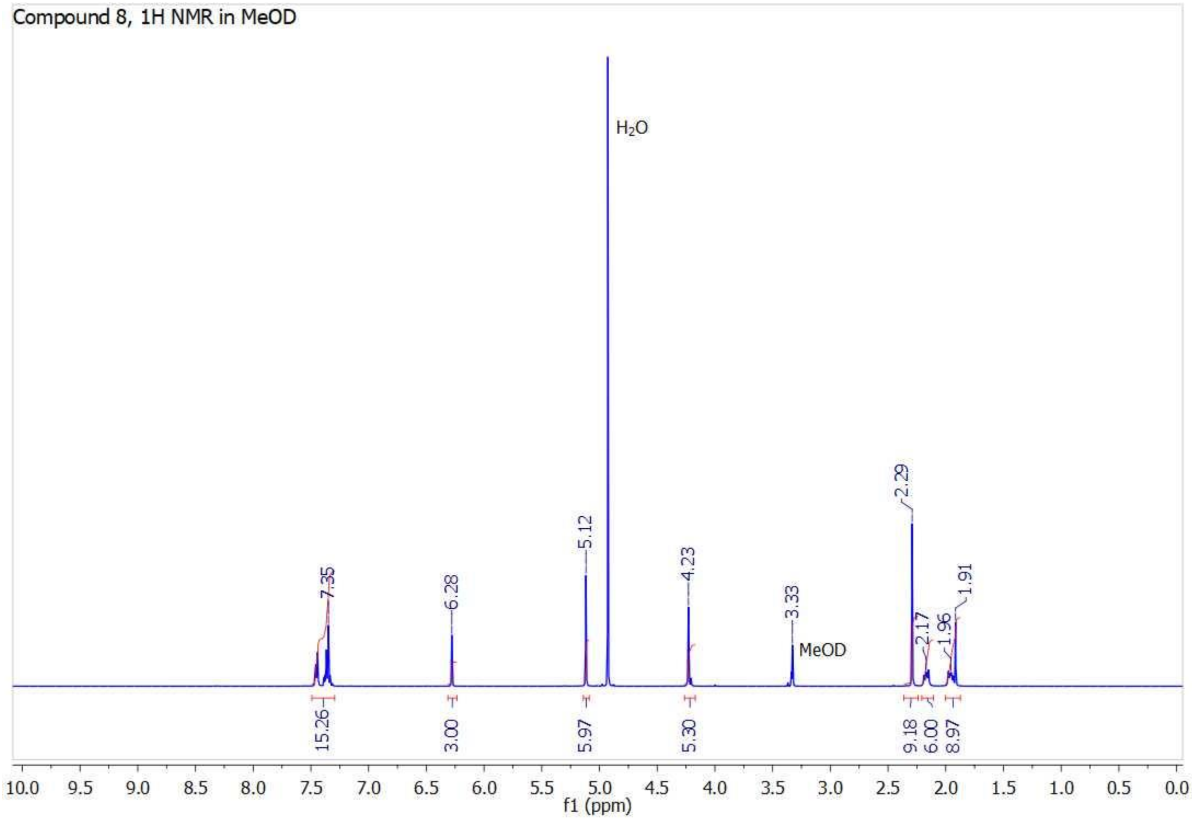

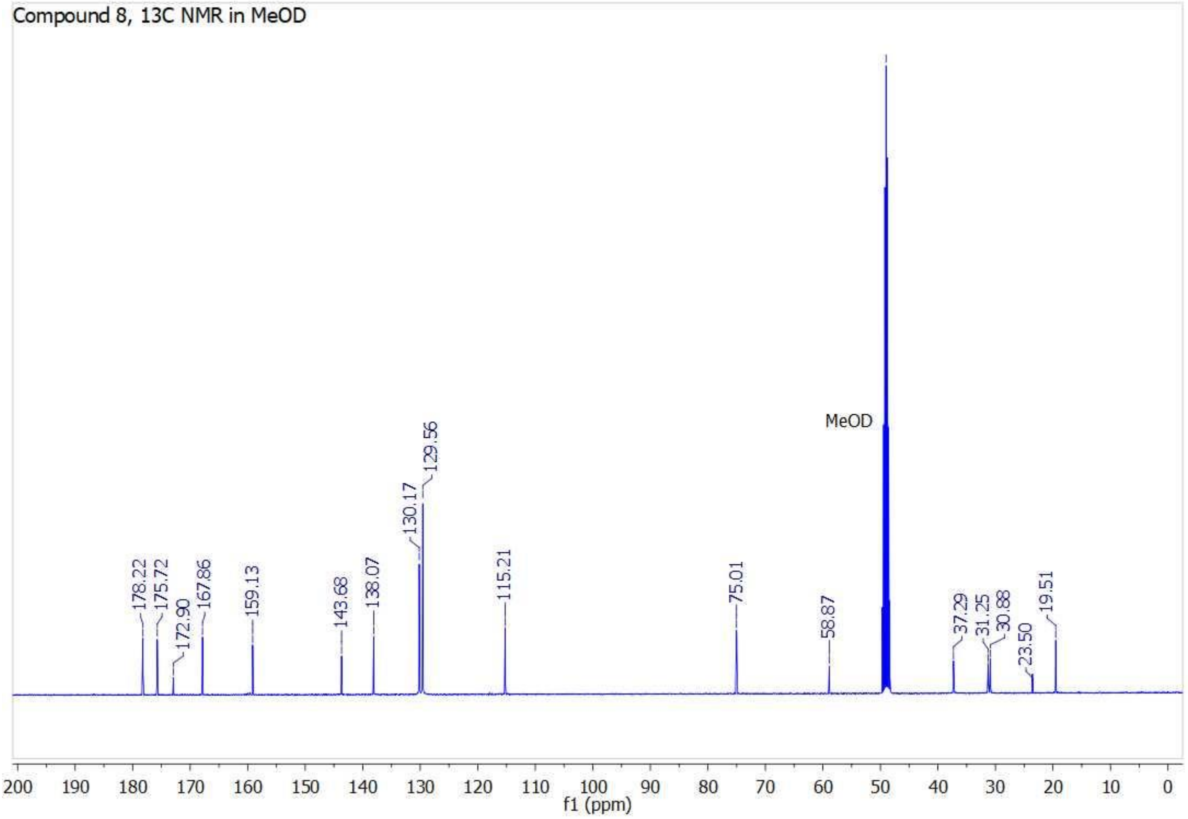

Figure S15. *^{1}H NMR (top panel) and ^{13}C NMR (bottom panel) of **8** in methanol-d4.*

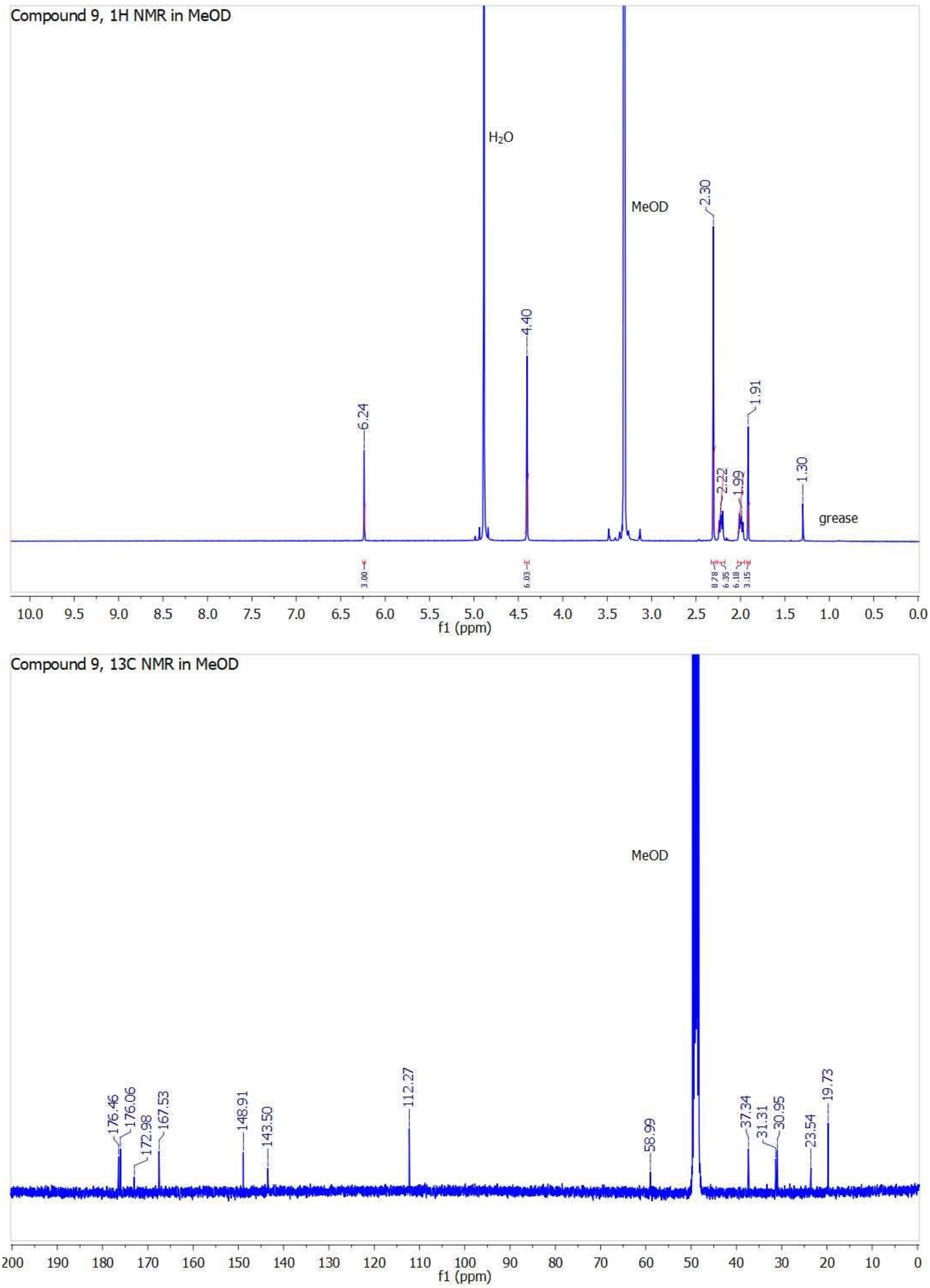

Figure S16. *^{1}H NMR (top panel) and ^{13}C NMR (bottom panel) of* ***9*** *in methanol-d4.*

References

1. S. Nawaz, PhD thesis, King's College London, 2013.
2. E. A. Martell and R. M. Smith, *Critical Stability Constants*, Plenum Press, New York, 1977-1989.
3. R. Cusnir, C. Imberti, P. J. Blower, M. T. Ma and R. C. Hider, *Int J Mol Sci*, 2017, 18, 116.
4. P. D. Taylor, *Talanta*, 1995, **42**, 845-850.
